# Transport limitations in polyolefin cracking at the single catalyst particle level†

**DOI:** 10.1039/d3sc03229a

**Published:** 2023-08-16

**Authors:** Sebastian Rejman, Ina Vollmer, Maximilian J. Werny, Eelco T. C. Vogt, Florian Meirer, Bert M. Weckhuysen

**Affiliations:** a Inorganic Chemistry and Catalysis, Institute for Sustainable and Circular Chemistry, Debye Institute for Nanomaterial Science, Department of Chemistry, Utrecht University Universiteitsweg 99 3584 CG Utrecht The Netherlands i.vollmer@uu.nl b.m.weckhuysen@uu.nl

## Abstract

Catalytic cracking is a promising approach to chemically recycle polyolefins by converting them into smaller hydrocarbons like naphtha, and important precursors of various platform chemicals, such as aromatics. Cracking catalysts, commonly used in the modern refinery and petrochemical industry, are tailored to process gaseous or liquid feedstock. Polyolefins, however, are very large macromolecules that form highly viscous melts at the temperatures required to break their backbone C–C bonds. Therefore, mass transport is expected to limit the performance of traditional cracking catalysts when applied to the conversion of polymers. In this work, we study these effects during the cracking of polypropylene (PP) over catalysts utilized in the fluid catalytic cracking (FCC) process. Thermogravimetric experiments using PP of varying molecular weight (*M*_w_) and catalysts of varying accessibility showed that low *M*_w_ model polymers can be cracked below 275 °C, while PP of higher *M*_w_ required a 150 °C higher temperature. We propose that this difference is linked to different degrees of mass transport limitations and investigated this at length scales ranging from milli- to nanometers, utilizing *in situ* optical microscopy and electron microscopy to inspect cut open catalyst–polymer composites. We identified the main cause of transport limitations as the significantly higher melt viscosity of high *M*_w_ polymers, which prohibits efficient catalyst–polymer contact. Additionally, the high *M*_w_ polymer does not enter the inner pore system of the catalyst particles, severely limiting utilization of the active sites located there. Our results demonstrate that utilizing low *M*_w_ polymers can lead to a significant overestimation of catalyst activity, and suggest that polyolefins might need to undergo a viscosity reducing pre-treatment in order to be cracked efficiently.

## Abstract 2

Catalytic cracking could enable low temperature conversion of hard-to recycle polyolefin plastics. However, traditional cracking catalysts suffer from macro and microscopic mass transport limitations, which call for plastic pre-treatment.

## Graphical abstract

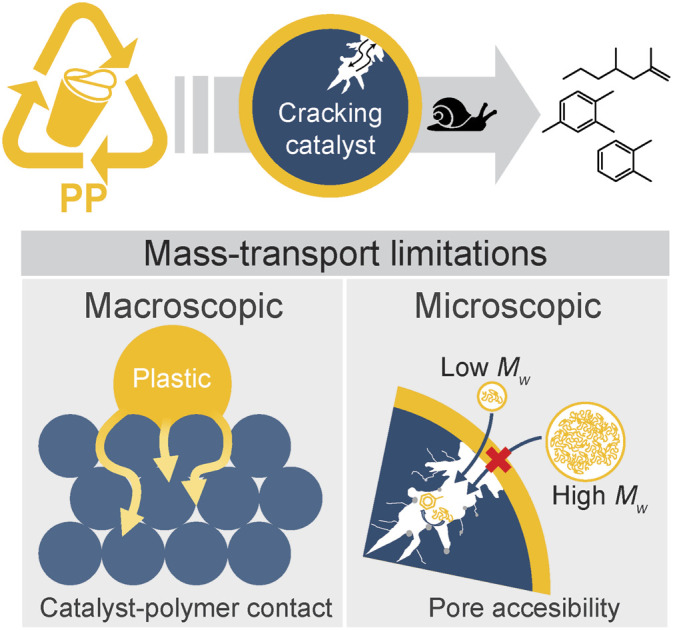

## Introduction

Traditional recycling of plastic waste mostly relies on melting and re-extrusion of well-sorted and purified plastic waste streams.^1^ However, due to contamination and progressive degradation of the polymer chains, the resulting product is generally of lower quality than the original virgin plastic.^2,3^ This significantly inhibits the increase of recycling volumes. To date, less than 10% of all plastic ever produced was recycled.^4^ Chemical recycling could help to improve recycling rates significantly. Unlike in conventional mechanical recycling, the plastic is converted into smaller chemical building blocks. The resulting products can then be used directly *e.g.* as fuels,^5^ cracker feed^6^ and lubricants,^7^ or alternatively further processed by existing chemical infrastructure to various products including polymers.^1,8^ A key advantage of chemical recycling is that the products can be of comparable quality to their crude oil-derived counterparts.

Feasible chemical recycling techniques strongly depend on the type of polymer to be processed. For polycondensates (*e.g.*, polyethylene terephthalate (PET), and Nylon), functional groups offer a chemical point of attack that can be utilized in solvolytical approaches to yield the corresponding monomers.^8,9^ For polyolefins, the most produced plastics,^4^ no such chemical points of attack are present. Instead, the C–C bonds that make up the polymer backbone have to be cleaved to yield smaller molecules. The simplest way to achieve this is by heating the polymer under an inert atmosphere in a process called pyrolysis. This technique is already applied commercially^1^ and can give high yields of monomers for polystyrene (PS)^10^ and polymethylmethacrylate (PMMA).^11^ For the most common polyolefins however, namely polyethylene (PE) and polypropylene (PP) a rather low value ‘pyrolysis oil’ consisting mostly of branched alkenes and cyclic alkanes is obtained.^12^ The main cause of this is the random-scission mechanism of the decomposition, which is mediated by radicals.^13^ Utilizing a catalyst for the conversion of polyolefins has been shown to lower the required reaction temperature,^14–19^ and therefore likely also the energy requirements of the process, as well as shift the product distribution to more valuable products, such as aromatics.^14^ If a catalyst is utilized, the process can be labelled catalytic cracking or catalytic pyrolysis. Depending on whether the catalyst in direct contact with the plastic (as is the case in the present study) or used to crack thermal pyrolysis vapours the terms ‘*in situ*’ or ‘*ex situ*’ catalytic pyrolysis are being used.^20^

The key advantages of catalytic cracking in comparison to other chemical recycling routes for polyolefins, *i.e.* hydrocracking and hydrogenolysis,^21,22^ is that it does not require high pressure hydrogen, can be achieved at shorter reaction times,^8^ and that continuous processes have been investigated.^23–25^

Research on catalytic cracking of polyolefins mostly focuses on utilizing traditional heterogeneous solid acid cracking catalysts from the petrochemical industry, like zeolites and silica/alumina.^8^ Zeolites for example, are used to convert fossil feedstock most comparable to molten plastic, that is vacuum gas oil (VGO), a high boiling, viscous fraction of crude oil with carbon numbers between 35 and 40.^26^ It is processed to smaller hydrocarbons like gasoline and propylene using a process called fluid catalytic cracking (FCC). This process is a key element in the modern petroleum refining infrastructure.^27^ The catalyst (further coined as FCC-cat) is generally composed of a spray-dried mixture of silica–alumina, clay and zeolite Y (FAU topology), which as a solid acid forms the principal active phase.^27,28^ The catalyst particles are generally 50–150 μm in size. During the FCC process, the catalyst is progressively poisoned by metal deposits originating from the feed and reactor walls, and the zeolite component is degraded due to the harsh regenerator conditions.^27^

**Fig. 1:**
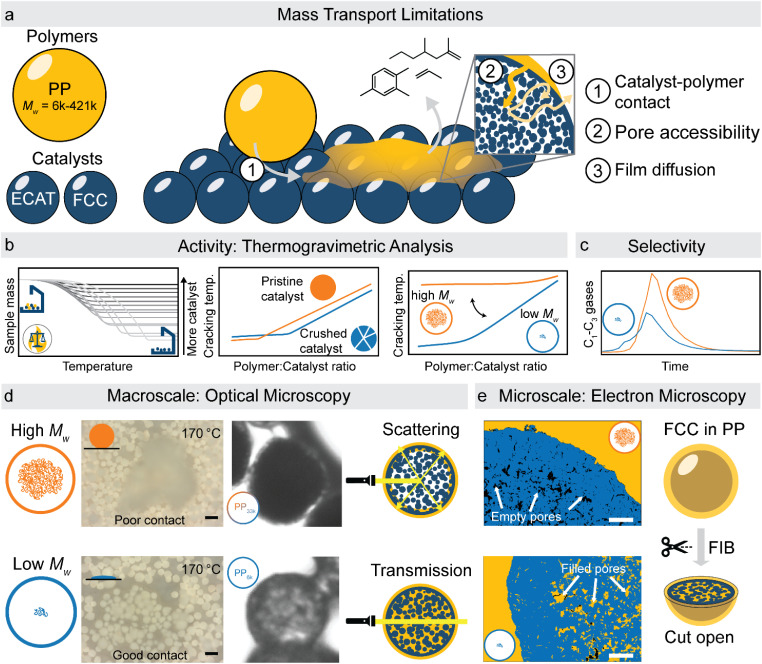
Summary of experimental approach utilized in this study. (a) Schematic representation of polymers and catalysts utilized as well as three types of transport limitations identified. (b) Catalyst activity was investigated using non-isothermal thermogravimetric analysis of catalyst : polymer mixtures. The effect of catalyst structure and polymer molecular weight were investigated by determining the required cracking temperature at different catalyst : polymer ratios. Lower cracking temperature corresponds to higher effective activity. (c) The effect of mass transport limitations on selectivity was studied using semi-batch reactor experiments with (on-line) gas chromatography. (d) Catalyst–polymer interactions on a macroscopic scale were investigated using *in situ* optical microscopy, allowing to identify differences in catalyst–polymer contact for polymers of different *M*_w_. Additionally, polymer entering inside the particle interior turns the particle more transparent to light by reducing scattering, allowing to qualitatively asses catalyst accessibility using simple optical microscopy. Scale bars: 100 μm. (e) On a microscale, electron microscopy of catalyst–polymer composites which were cut-open using a focussed ion beam allows to directly determine the accessibility of the catalyst pores. Micrographs were segmented for clarity. Scale bars: 3 μm.

This requires continuous catalyst replacement, in some cases up to 30 tons per unit per day.^27^ Catalyst removed from the unit is termed equilibrium catalyst (further coined as ECAT). ECAT is sold to other refineries, can be used in construction,^29^ or landfilled. It is available in large quantities,^27^ making it attractive in the use of plastic waste cracking.^14^ However, utilizing these and other traditional solid acid catalysts for polymer cracking introduces several challenges. Plastic melts behave very differently to gases and liquids, which are the traditional reactants in cracking catalysis. They are solid at room temperature and highly viscous when molten.^30^ The fluidized bed reactors, which are used in the FCC process, suffer from defluidization when used to process plastic, as the molten polymer glues catalyst particles together.^31^ In addition, polymer macromolecules are orders of magnitude larger than the fossil-based hydrocarbons processed using these catalysts. The molecules in the VGO feedstock, already the largest molecules processed catalytically in industry, have an average diameter of 3.5 nm (determined by size-exclusion chromatography).^26^ For comparison: the average chain lengths of PP of a weight averaged molecular weight (*M*_w_) of ∼23 000 g mol^−1^, and ∼307 000 g mol^−1^ (from now on referred to as PP_23k_ and PP_307k_), are 140 nm and ∼1.86 μm respectively, corresponding to strongly idealized random coil diameters of 13 nm and 46 nm (r.f. S1† for the corresponding calculations). This comparison raises the question how well these polymer macromolecules can enter the particle pore system. For reference it is important to recall that commercial PP homopolymer used in packaging can have a *M*_w_ between 141 000 g mol^−1^ and 642 000 g mol^−1^ (see ESI S2† for details). On one hand, a study suggested that high-density polyethylene can enter pores as thin as 1.5 nm in diameter if dissolved in dichlorobenzene and heated at 130 °C for 6 h.^32^ However, unlike in dilute solution, the polymer chains are still entangled in the molten state. If the chains first need to be untangled before they can enter the pore network, the process can be expected to take even longer, while coils are likely too large to enter the narrow channels of *e.g.*, zeolites. Therefore, polymers can be expected to not enter catalysts designed for fossil feedstocks efficiently. Significant mass transport limitations can be expected – holding back the development of effective catalysts that could enable the conversion of large volumes of plastic waste to valuable chemicals at mild conditions. In heterogeneous catalysis, one speaks of transport limitations when the activity of the catalyst is not inhibited by the nature of the active site itself, but rather by insufficiently fast transport of molecules to and from the active site. In this context, the classical “7 steps of heterogeneous catalysis”, reported in catalysis textbooks, extend the simplified picture of adsorption-reaction-desorption by considering film and pore diffusion.^33^ For polyolefin cracking, an additional step might also be added: macroscopic contact of polymer and catalyst (see Fig. 1a). It must be kept in mind though, that the physical processes dictating the initial entering of reactants into the pores of the catalysts differ from gas or liquid phase catalysis. In solution or gas phase, entering of reactants into the catalysts is described by diffusion. For polymer melts and higher hydrocarbons contacting porous systems, capillary intrusion described by Washburn's or related equations determines entering of polymers or higher hydrocarbons into the pores.^34–37^ While several prior studies refer to transport phenomena as a limiting factor,^14–19^ systematic studies with a focus on gaining insights for catalyst development are, to the best of our knowledge, lacking, although a recent study investigated mass transport of co-reactants in polymer hydrocracking.^38^ In this work, we systematically study the effects of transport limitations for the cracking of PP over fluid catalytic cracking catalysts (FCC-cat) by evaluating their activity and studying polymer–catalyst interaction from mm to nm length scales.

In prior work, our group showed that applying a spent FCC catalyst (ECAT) to polyolefin cracking yields an aromatic rich hydrocarbon product and significantly less coking when compared to a fresh catalyst.^14^ Its hierarchal pore structure, subject to many studies in the past,^39–42^ makes it an interesting model catalyst to investigate transport limitations. It is important to mention here that we drew inspiration from multiple prior studies. Already in 1996, Liu *et al.* noted that tetradecane can be catalytically cracked at a 200 °C lower temperature than PE and attributed this to mass-transport limitations.^15^ By investigating a broader range of molecular weights, a more precise picture on the impact of transport effects could be drawn. A key technique for this type of activity studies is thermogravimetric analysis (TGA), a common method in the study of the apparent kinetics of polymer degradation.^16,18,19,43–47^ In short, the sample is heated while precisely measuring its weight. Since cracking of polymers generates gases and therefore leads to a drop in sample mass, it can be used to determine the required cracking temperature with high accuracy and allows to quickly compare the activity of different catalysts. Serrano *et al.* used TGA experiments to show that the cracking activity of the zeolite ZSM-5 directly depends on the external surface area of catalyst crystallites.^18^ The fact that accessibility is likely key to catalyst activity in plastic cracking has been utilized to compare the accessibility of zeolites that have been desilicated to a different degree.^48^ Other authors have compared catalysts of different accessibilities, for example by comparing the activity of zeolite-Y, which has a pore size of 7.4 Å with ZSM-5, which has a pore size of 5.4–5.6 Å,^49^ or by comparing zeolites to mesoporous materials.^50^ However, in these comparisons accessibility is not the only factor changed. Accessibility related effects can therefore not be disentangled from other parameters affecting the activity, for example acid site concentration and strength. It is therefore necessary to compare catalysts where accessibility is the only property that changed. Finally, multiple studies have noted that the catalyst–polymer ratio in this type of experiments has big impact on the required cracking temperature. Manos *et al.* therefore proposed that initial cracking only occurs at the outer surface of zeolite crystals.^16^ The dependence of the required temperature on the polymer : catalyst ratio was also utilized to gain deeper insight into the cracking kinetics *via* kinetic modelling.^19,46^

Herein we have combined the multiple approaches listed above to the catalyst systems under study. By conducting TGA experiments with polymers of different molecular weights, at different polymer : catalyst ratios, comparing catalyst where only the accessibility is changed, and utilizing kinetic modelling, different types of transport limitations could be identified (Fig. 1b). To gain insight into which physical properties of polymer and catalyst are responsible for these transport limitations, we have utilized two new approaches to study catalyst–polymer interactions: *in situ* optical microscopy of the cracking reaction was used to study effects at the mm–μm scale (Fig. 1d), while focused ion beam-scanning electron microscopy (FIB-SEM) of polymer–catalyst composites allowed to study effects in the μm–nm range (Fig. 1e). To evaluate the effect transport limitations have on reaction selectivity, semi-batch pyrolysis experiments were utilized (Fig. 1c). Together, these results allow for a better understanding of the mass-transport limitations in the catalytic cracking of plastics and could allow to identify key design criteria for new catalysts enabling milder conditions and higher selectivity.

## Results and discussion

### Effect of polymer : catalyst ratio on cracking kinetics

**Fig. 2:**
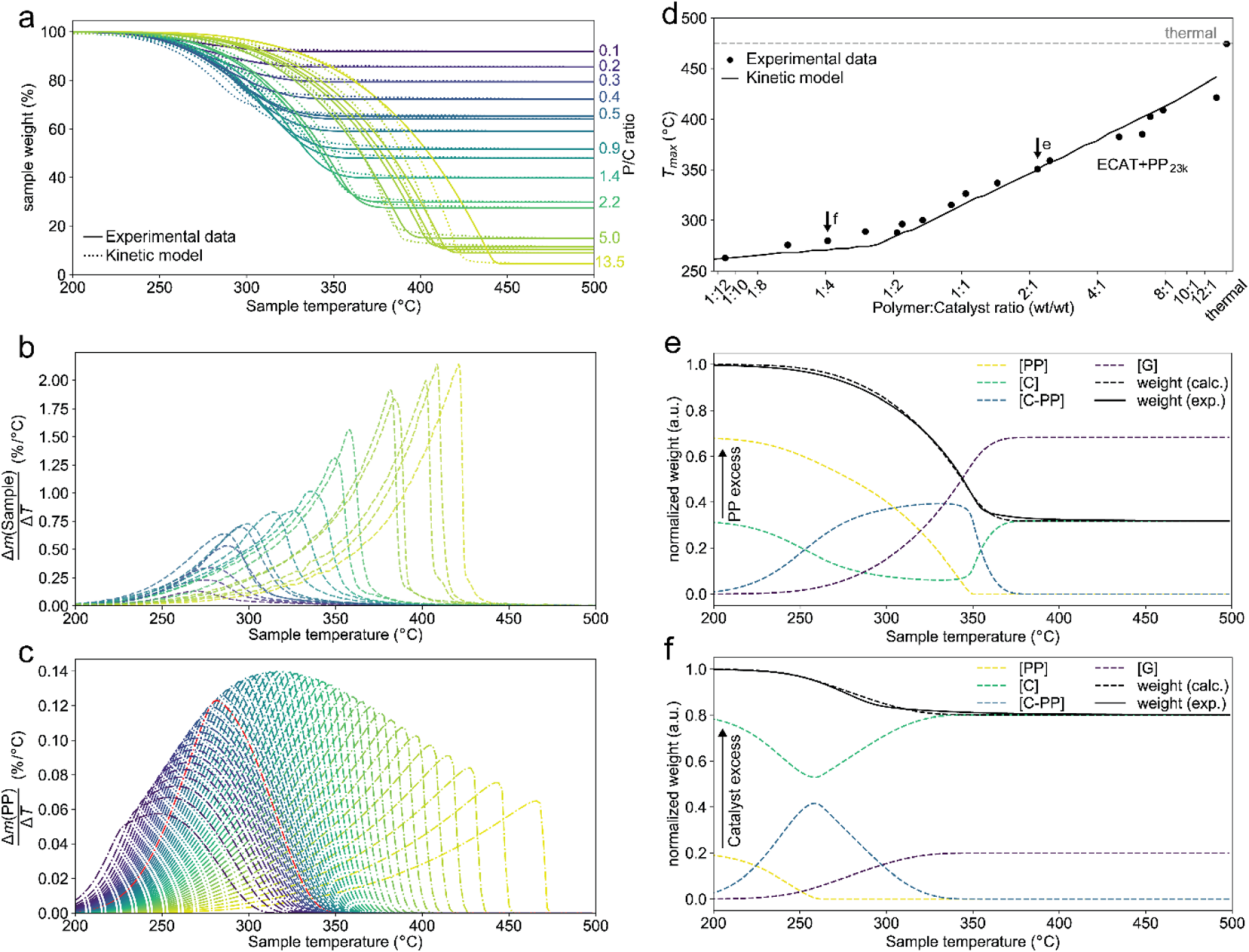
Results of thermogravimetric analysis (TGA) of PP_23k_ cracking using ECAT at various polymer : catalyst (P : C) ratios and kinetic modelling. Heating rate: 10 °C min^−1^. (a) TGA profiles and fit of the kinetic model. (b) Derivative TGA profiles in units of total sample weight. (c) Derivative TGA profiles for various P : C ratios predicted by the kinetic model in units of polymer weight. P : C increases from blue to yellow, where pw denotes normalized polymer weight. (d) Plot of the temperatures at which the decomposition rate is highest (*T*_max_) for cracking of PP_23k_ with ECAT at various P : C ratios as determined experimentally and extrapolated by kinetic modelling. Arrows indicate data points used in the panels below. (e and f) Development of cracking species concentrations determined by kinetic modelling for a P : C ratio of ∼2 : 1 (e), and ∼1 : 4 (f).

In a typical TGA experiment, catalyst and polymer were mixed at polymer : catalyst (P : C) mass ratios from ≈12 : 1 to ≈1 : 12, and heated under nitrogen atmosphere to crack the plastic. For a justification of the approach the reader is referred to the ESI (S3).†Fig. 2a shows the TGA cracking profiles for the combination of ECAT with PP_23k_ (*M*_w_ determined by GPC, see Fig. S1 and Table S1†), while Fig. 2b shows the derivatives of the respective mass–loss curves. It is evident that the P : C ratio had a large effect on the reaction rate. The temperature at which the decomposition rate is the highest (*T*_max_) can be found as the maximum in the derivative TGA (Fig. 1b). For easier comparison *T*_max_ is shown in relation to the P : C ratio (Fig. 2d).

While for thermal pyrolysis *T*_max_ was ∼475 °C, the temperature dropped to ∼400 °C with the addition of a small amount of catalyst. *T*_max_ then continuously dropped with a decreasing P : C ratio, as also noted previously.^19^ For P : C ratios below 1 : 4, *T*_max_ dropped noticeably slower with decreasing P : C, and reached temperatures below 275 °C for P : C < 1 : 6. This shows that PP cracking can be conducted at a 200 °C lower temperature than for purely thermal pyrolysis. It has to be noted, that in the FCC process similarly large excesses of catalyst material are common,^27^ and a large excess of catalyst material being required to crack PP at low temperature has also been noted previously.^17^ The drop in *T*_max_ with increasing catalyst loading can be captured by a kinetic model, where the catalyst enters into the rate equation (Fig. 1c and d).^19^ In a first step, [PP] and the catalyst [C] form a polymer–catalyst complex, which is released upon gaseous product formation [G], where *c* is a proportionality constant describing the mass ratio of PP to C in the polymer–catalyst complex (eqn (1)).1
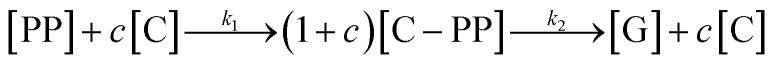


The gaseous product formation corresponds to the weight loss observed in TGA. The corresponding rate equations given in mass-concentrations, adjusted from Marcilla *et al.*,^19^ are:2
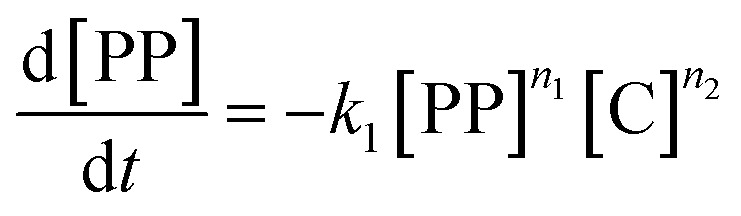
3
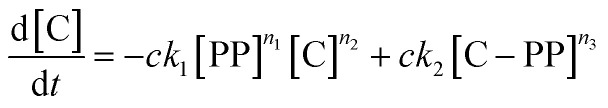
4
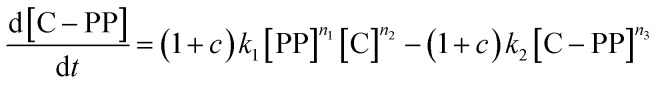
5
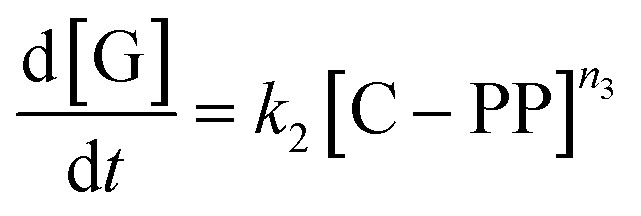
6
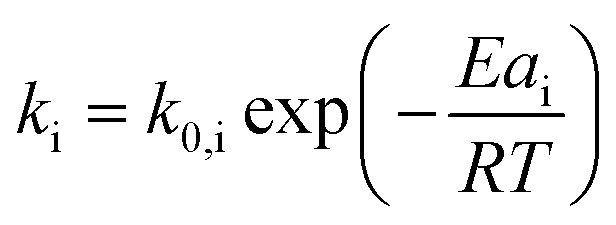
*n*_1_–*n*_4_ are apparent reaction orders in mass concentrations.

This model fits the experimental data satisfactorily (Fig. 2a), especially for intermediate P : C ratios. At low P : C ratios, coking is expected to also influence the shape of the weight loss curve, as active sites become increasingly blocked. This creates a mismatch with the model at a higher conversion, while at high P : C ratios some PP is expected to be too far away from any catalyst surface to interact at all and therefore undergo thermal cracking. The trend in *T*_max_, however, is captured excellently by the applied model, except at the highest P : C ratio (Fig. 2d). As more catalyst material and therefore active sites are added, the reaction rate increases, and the temperature at which the reaction rate is the highest drops. Interestingly, the rate of [C–PP] build-up is more dependent on [C] than [PP] as the reaction order (*n*_2_ = 1.81) of [C] is higher than that of [PP] (*n*_1_ = 0.52) and thus the rate is directly proportional to [C]. Reaction orders > 1 in catalyst active site concentration have been observed in hexane cracking and have been attributed to a mechanism involving multiple active sites.^51^ At P : C ratios above 1 : 2, the reaction enters an active site depleted regime and the catalyst complex [C–PP] is depleted at the same rate at which it is formed. This can be seen from the development of species with temperature (Fig. 2e). For high P : C ratio a crossover point of the [PP] and [C–PP] concentrations is observed, which means that build-up and depletion rates of [C–PP] are similar. At P : C ratios below 1 : 2, the build-up of [C–PP] is faster and happens earlier and the weight loss rate is mostly determined by the depletion rate of [C–PP], which only starts after all [PP] has reacted to the intermediate (Fig. 2f). The activation energy barrier of [C–PP] depletion is higher than that of [C–PP] formation (Table S3†), while the pre-exponential factor is higher for depletion. This means that the rate constant *k*_1_ increases faster with temperature than *k*_2_ (Fig. S3†). *k*_1_ also decreases very fast below 300 °C. This can be identified as the kinetically limited regime and leads to a shift in the skewing of the differential thermal gravimetric analysis (DTGA) profile from right-centred to Gaussian and finally to left-centred (Fig. 1c). The transition from an active site depleted regime to a kinetically limited regime can also be observed as the ‘elbow’ in Fig. 1d at P : C ≈ 1 : 2 (Fig. 2d). While an active site depleted regime could be explained by a lack of active sites on the catalyst, it could also be evidence of significant accessibility and mass transport limitations. We suspect that the concentration of accessible active sites is significantly lower. The effective active site concentration could be lowered by micro- and/or macroscopic effects (Fig. 1a).

(i) Microscopic: at the length scale of the catalyst pore diameter (nm to few μm), large polymer macromolecules might not enter the pore system. This could lead to only a fraction of the particles' active sites being utilized. We refer to these effects as accessibility-related. In this regime, only part of the catalyst particle participates in the reaction leading to a low catalyst effectiveness.

(ii) Macroscopic: on the length scale of a whole catalyst particle (50–150 μm), the high viscosity of the polymer melt could lead to insufficient contact of polymer with external particle surface. The zero-shear viscosity (*η*_0_) of PP melts scales with the molecular weight according to eqn (7).^52^

7 *η*_0_ ∝ *M*_w_^3.4^

For high *M*_w_ polymers, some particles might therefore not participate in the reaction at all.

### Pore accessibility

**Fig. 3:**
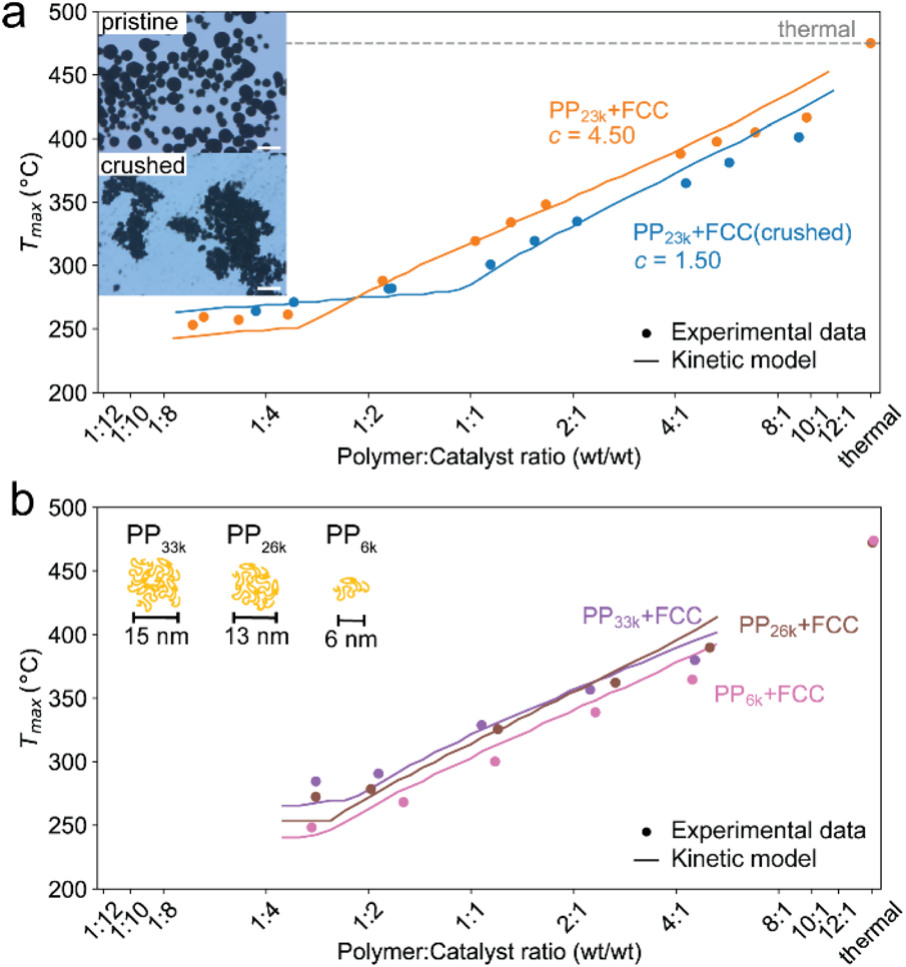
(a) *T*_max_ for cracking of PP_23k_ using FCC-cat and the same catalyst crushed before reaction at various P : C ratios. Trendlines obtained by kinetic modelling. *c* is a proportionality constant describing the mass ratio of PP to C in the polymer–catalyst complex. Inset: optical microscopy images of pristine and crushed FCC-cat. The scale bars correspond to 100 μm. (b) *T*_max_ for cracking of PP resins of different *M*_w_ at various P : C ratios. Inset: schematic representation of random coil sizes for polymers studied. For full TGA profiles we refer to Fig. S4 and S5.†

**Fig. 4:**
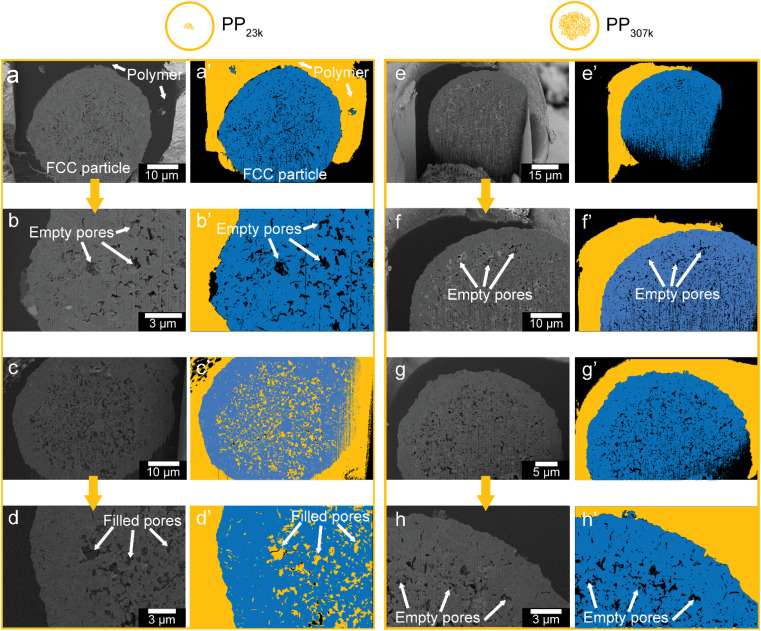
Scanning electron microscopy (SEM) images acquired in backscattered electron mode (BSE) displaying cross-sections of FCC particles embedded in polymer, cut open with a focused ion beam. The vertical stripes that appear in some of the images originate from the FIB cutting procedure and are not inherent to the studied particles. (a–d) FCC-cat in PP_23k_. (a′–d′) Segmented images, polymer depicted in yellow, catalyst in blue. To guarantee a correct segmentation of the pores, certain grey scale values were not allocated to the polymer or the support phase. (e–h) FCC-cat in PP_307k_. (e′–h′) Segmented images.

To test for accessibility problems, the PP_23k_ cracking activity of a pristine fresh FCC-cat was compared to the activity of the same catalyst that was crushed before reaction (Fig. 3a). This treatment lowers the particle size significantly and should improve accessibility to active sites previously embedded deep in the particle. The crushing did not lower the BET surface area (Table S2 and Fig. S6†). At P : C ratios higher than 1 : 2, *T*_max_ is ∼25 °C lower for the crushed catalyst than for the pristine one, demonstrating a significantly higher activity. At large excess of catalyst, however, *T*_max_ is similar for both samples. We interpret this as reaching of the kinetic regime, at which *T*_max_ is more affected by the activation energy than by the effective active site concentration (*vide supra*). The improved activity suggests that the reaction mostly takes place at the outer particle surface and that the number of accessible active sites is severely limited on the pristine catalyst. However, experiments utilizing different size fractions of the same catalyst obtained by sieving showed that the activity is not significantly affected by particle size, and the activity of an ECAT is similarly not affected by crushing or sieving (Fig. S7†). Depending on preparation technology, FCC catalysts can contain a dense outer ‘shell’, that grants the particles a higher mechanical stability, at cost of accessibility.^40,53^ The FCC catalyst under study contains such a ‘shell’ (see Fig. 4).

**Fig. 5:**
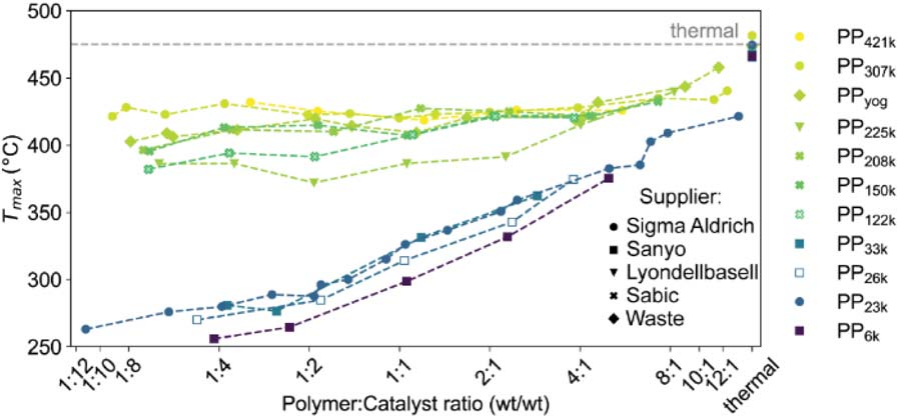
*T*_max_ for cracking of various PP samples obtained from different manufacturers using ECAT at various P : C ratios. For full thermogravimetric analysis (TGA) profiles we refer to Fig. S4 and S5.†

In the case of the crushed catalyst, polymer does not always have to travel through the dense shell to reach active sites on the particle interior, enabling more active sites to participate in the reaction. The lack of improved activity upon crushing for the ECAT can be explained by the lack of a thick, dense ‘shell’ (see Fig. S8†) and therefore improved accessibility for this catalyst. An alternative explanation might be that due to the overall smaller amount of acid sites on the ECAT,^14^ the improved accessibility has a smaller effect. Differences in catalyst effectiveness can also be demonstrated using kinetic modelling. The kinetic model demonstrated in Fig. 2 was applied to both datasets depicted in Fig. 3a by first optimizing parameters for the crushed catalysts, and consecutively optimizing only parameter *c* (see eqn (1)–(4)) for the pristine catalyst. This parameter can be interpreted as an ‘effectiveness factor’: for a lower *c*, less catalyst is required to crack a unit of polymer, lowering in *T*_max_ and shifting the described kinetic regime towards higher P : C. For the crushed catalyst, *c* is significantly lower. As the remaining parameters, *e.g.*, activation energies, are kept fixed, the increase in activity can be attributed to an improvement in catalyst utilization. Accessibility problems are also evident when the cracking activity of FCC and ECAT is compared for PP samples of different *M*_w_ (Fig. 3b and 5). *T*_max_ drops with decreasing *M*_w_ across a wide range of P : C ratios. While for FCC the trend is very slight and a clear difference is only seen when comparing PP_33k_ and PP_26k_ to PP_6k_, it is slightly more evident for ECAT when only Sanyo polymers are compared. Furthermore, *T*_max_ is very reproducible for a given catalyst–polymer combination if the same P : C ratio is hit (see Fig. S2a, S7a and b†). This suggests that the particle pore network is less accessible to the larger macromolecules.

To collect further evidence for accessibility limitations and to investigate how far PP penetrates inside the FCC particle interior, electron microscopy was utilized. First, PP samples (PP_23k_ and PP_307k_) were placed in vials and heated to 180 °C. This temperature is above the melting points (158 °C for PP_23k_, 172 °C for PP_307k_, determined by differential scanning calorimetry (DSC), Fig. S8†) but below the minimal reaction temperature to prevent cracking to smaller molecules (Fig. S4†). FCC-cat was added, and the mixture briefly stirred. After 20 min, the resulting composites were removed from the vial and cooled. Particles of the composite including completely embedded FCC-cat particles were cut out using a scalpel and transferred to a scanning electron microscope (SEM) equipped with a focused ion beam (FIB). Following a method adopted by our group for similar composite systems,^54,55^ the composite particles were cut open to reveal cross sections of FCC particles embedded in polymer (Fig. 4). The resulting images were segmented using manually set thresholds to distinguish between catalyst, polymer, and empty pores. In total, 2 particles per polymer were analysed. While due to its involved nature this analysis cannot provide statistics about accessibility, it still can serve as an example of the poor accessibility of the FCC particles to polymer melts. For PP_23k_, one particle showed no penetration of polymer into the particle interior, as can be seen in the raw (Fig. 4a and b) and segmented images (Fig. 4a′ and b′). In the other particle, however, the majority of the interior pores are filled with polymer (Fig. 4c and d′). This shows that already for the low *M*_w_ polymer pore transport into the particle interior can be significantly hampered, and the reaction can be expected to proceed to a large extent on the particle's exterior surface. This is consistent with a prior study that utilized confocal fluorescence microscopy (CFM) to show an accumulation of aromatic species primarily in a ring at the outer particle surface, while no fluorescent species were found in the particle interior.^14^ For PP_307k_ no plastic was found inside the particle interior for both analysed particles (Fig. 4e–h′), indicating that pore utilization for higher *M*_w_ plastic is lower. More statistically significant information further indicating poor accessibility even for PP_33k_ was obtained by *in situ* optical microscopy (*vide infra*, Fig. 7). Insufficient accessibility of pores is difficult to attribute to a single effect or property. If very large pores in the μm range are considered, the melt viscosity can be expected to play a key role. According to Washburn's equation, the rate of capillary intrusion scales with the inverse viscosity.^56^ As the viscosity of PP melts drops with increasing temperature according to an Arrhenius-type equation,^57^ the polymer is going to intrude faster at higher temperature and accessibility will likely be improved. When pores in the nm or Å size range are considered, steric effects are likely determining mass transport. For all pore diameters in-between these two limits, a variety of other effects also need to be considered, including *e.g.*, self-diffusion^58^ and capillary forces. While studies have shown that the Lucas–Washburn equation describing capillary filling can be used to model the intrusion of polymer melts and higher hydrocarbons in porous systems,^34–36,59^ it is difficult to assess which physical effects precisely are the limiting factor from the results of this study.

### Catalyst–polymer contact

**Fig. 6:**
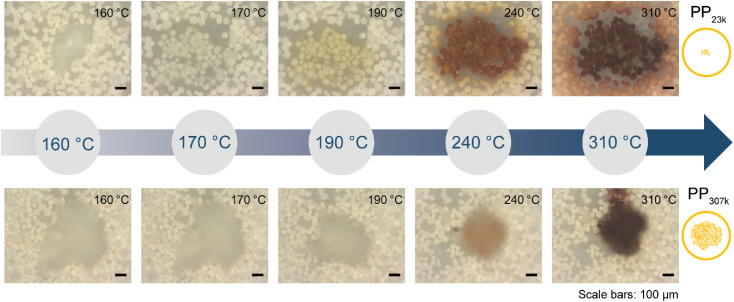
*In situ* optical microscopy of polypropylene (PP) cracking using FCC catalyst. Individual polymer grains were placed on a bed of FCC catalyst and heated at 10 °C min^−1^ under N_2_ flow in a Linkam stage. Images were acquired every minute. See ESI† for videos of the full reaction. Top: PP_23k_. Bottom: PP_307k._

To test for limitations caused by insufficient catalyst–polymer contact, further TGA experiments using ECAT as catalyst with PP of various *M*_w_ obtained from different suppliers including high *M*_w_ polymers which are commercially applied were conducted. A large increase in *M*_w_ should lead to significantly higher melt viscosity (eqn (7)). In addition, cracking of PP derived from a yoghurt cup (PP_yog_) was studied to determine to what extent cracking of realistic PP waste is affected by this type of transport limitation (Fig. 5). All polymers investigated are more than 80% isotactic (see Table S1 and Fig. S10†). The polymers investigated can be divided into two groups. Polymers with *M*_w_ of 122 000 g mol^−1^ and above (now collectively referred to as ‘high *M*_w_’) behaved very differently compared to PP of lower *M*_w_. For all low *M*_w_ polymers, *T*_max_ drops significantly below 300 °C in large excess of catalyst, while for the high *M*_w_ polymers *T*_max_ does not drop below ∼375 °C even in large excess of catalyst. While all high *M*_w_ polymers show an initial drop in *T*_max_ with increasing catalyst concentration, the cracking temperature equilibrates and does not drop significantly further, suggesting a different type of limitation then observed for the low *M*_w_ polymers. For the highest *M*_w_ PPs investigated (PP_307k_, PP_421k_) *T*_max_ remains constant around 425 °C for a broad range of P : C ratios, suggesting the existence of a *M*_w_ beyond which a further increase no longer impacts the required cracking temperature. For PP_225k_–PP_122k_ a trend can only be identified if polymers from the same manufacturer are compared. A decrease in *M*_w_ leads to a decrease in cracking temperature for PP_208k_–PP_122k_. Surprisingly, PP_225k_ shows a lower cracking temperature than PP_122k_, suggesting that a polymer property other than molecular weight, *e.g.*, degree of long chain branching or additives added by the manufacturer, are influencing cracking behaviour. Macroscopic particle size did not significantly affect the cracking temperature, as control experiments with melted down polymer showed (Fig. S11†). Differences in molecular weight distribution might have an effect on cracking behaviour, however a study suggested that for PS solutions dispersity only has a minor influence on zero-shear viscosity.^60^*T*_max_ for PP_yog_ behaves similar to the other high *M*_w_ polymers investigated. To investigate whether limited contact due to higher melt viscosity is responsible for the dramatic increase in required cracking temperature, we have conducted *in situ* optical microscopy of the cracking reaction. Individual grains of PP were placed on a bed of fresh FCC catalyst in a Linkam stage under constant N_2_ flow. The temperature was ramped at 10 °C min^−1^ and micrographs were acquired every minute until the temperature reached 500 °C. Fig. 6 depicts a selection of acquired images for the reaction of both PP_23k_ and PP_307k_. Full movies of the reactions can be found in the ESI.† Up to a temperature of 160 °C no changes were observed for both samples. However, once the melting point (156 °C for PP_23k_, 172 °C for PP_307k_, determined by DSC) was crossed, the samples behaved very differently. The low *M*_w_ PP_23k_ flowed instantly into the catalyst bed. Catalyst–polymer contact appears not to pose a significant problem for the low *M*_w_ plastic. On the other hand, PP_307k_ did not flow into the catalyst bed. Instead, the plastic particle slowly formed into a sphere. The only point of contact of polymer and catalyst was at the interface of the molten polymer sphere and the particles that it touched. We attribute this to the significantly higher melt viscosity of the sample. The particles also changed colour over the course of the reaction. For PP_23k_, a colour change of the FCC-cat particles from white to yellow was observed at 190 °C, indicating that the formation of aromatic products begins already at this low temperature. A slight colour change from clear to yellow was also observed for PP_307k_ at 220 °C (see Fig. S12†). This suggests that the activation barrier for aromatic formation of both samples is similar, as the associated colour change occurs at similar temperatures. At a temperature of 240 °C the catalyst particles in the PP_23k_ sample were of a brown colour associated with coke formation, and lost their glossy appearance, suggesting that most of the plastic had been absorbed into the particles. Simultaneously, catalyst particles that had no immediate contact with the molten polymer began to change colour. This colour change spreads outwards with increasing temperature. We interpret this as a visual indication of hydrocarbon vapor formation. Gaseous cracking products diffuse to the neighbouring particles, where they are cracked further to smaller hydrocarbons and aromatics/coke that are responsible for the observed change in colour. This can be further confirmed by TGA: at large excess of catalyst, significant gas evolution began at comparable temperatures (Fig. S4†). In the same temperature range for PP_307k_, the catalyst particles in contact with the polymer sphere turned from yellow to brown.

Only at 310 °C a flow of molten polymer into the catalyst bed was evident for the high *M*_w_ sample, however at this point the catalyst particles beneath the melt droplet have turned to a brown colour, indicating the reaction already proceeded significantly at the interface. In the temperature range above 310 °C, the main change observed for PP_23k_ was an increasing change of colour for the FCC-cat particles from brown to black associated with coke formation. For the high molecular weight PP_307k_ sample the colour change of surrounding particles, associated with significant hydrocarbon vapor formation, began at 330 °C (see videos in ESI†), significantly later than for the low *M*_w_ polymer. *In situ* optical microscopy under identical conditions was also performed for PP_6k_, PP_33k_, PP_122k_, PP_150k_, and PP_225k_ (Fig. S13†). The polymers with *M*_w_ above 33 000 g mol^−1^ behaved similarly to PP_307k_, and analogously polymers with *M*_w_ of 33 000 g mol^−1^ behaved similarly to PP_23k_, in-line with results obtained by TGA (Fig. 5). Catalyst–polymer contact below 310 °C is very poor for the high *M*_w_ samples, showing that the number of active sites available for the high *M*_w_ polymers at lower temperatures is significantly lower. The insufficient availability of active sites leads to a drastically lower reaction rate, inhibiting efficient cracking. These observations do not fully explain the observed significant change in reactivity when PP_33k_ is compared to PP_122k_, or the lack thereof for PP_421k_ compared to PP_307k_. It is evident that a low melt viscosity is critical to ensure good catalyst–polymer contact which allows for cracking at temperature < 300 °C. The viscosity of a PP melt scales with *M*_w_^3.4^ (eqn (7)),^52^ and decreases with increasing temperature following a Arrhenius-type equation.^57^ If these two relationships are combined, the necessary increase in temperature to achieve a similar viscosity for a polymer of higher *M*_w_ can be coarsely estimated (Fig. S14†). For example, to match the viscosity of a given PP melt at 200 °C, a melt of a PP of twice larger *M*_w_ requires an estimated temperature that is ≈130 °C higher. If these relations are applied to the polymers investigated here, it becomes clear that *e.g.*, PP_307k_ cannot reach the viscosity of PP_23k_ in the temperature range investigated, unless the molecular weight is reduced through thermal or catalytic pre-cracking. Thermal cracking has been shown to affect temperature–viscosity relationships of PP at temperatures as low as 270 °C.^57^ Since the thermal cracking occurs at very similar temperatures for all polymers investigated (Fig. S5†) we conclude that the similar cracking behaviour for the highest *M*_w_ polymers is caused by the similar temperature range in which thermal degradation or ‘pre’-cracking occurs, which leads to a reduction in *M*_w_ and therefore viscosity. In contrast, for the low *M*_w_ polymers it appears that no thermal or catalytic pre-cracking is required to reach sufficient macroscopic catalyst–polymer contact. Note that pre-cracking will also improve the rate of capillary intrusion.

The results offer a different explanation for the conclusion of a recent study that found that HDPE can enter into ZSM-5 micropores.^61^ At the temperatures utilized in the study, pre-cracking could cause the decrease in *M*_w_ and the formation of small molecules, which can enter micropores more easily.

The question arises, in what way observations obtained from experiments without stirring (both TGA and *in situ* optical microscopy) are applicable to stirred systems. On one hand, the macroscopic contact between the polymer and the external surface of the catalyst particles (that is, not in the particle pores) will be improved by stirring. However, in our semi-batch reactor experiments, stirring did not lead to a significantly earlier light gas evolution, unlike a significant decrease in *M*_w_ (*vide infra*). According to Washburn's equation the penetration length *L* scales with the fluid viscosity *η* according to: *L* ∝ *η*^−0.5^. Therefore, the viscosity is not only critical for macroscopic contact in an unstirred system, but also for entering of the polymer into the particle interior. Therefore, we believe that the conclusions drawn from non-stirred model experiments are still relevant for unstirred systems. To significantly decrease the viscosity of the polymer by stirring, very high shear rates are required.^62^ It is unclear whether these high shear rates are easily achieved using a conventional stirred reactor, as opposed to *e.g.* an extruder. Further studies will be necessary to establish whether stirring can indeed significantly improve reaction rates by decreasing viscosity.

### Using optical microscopy to probe pore transport

**Fig. 7:**
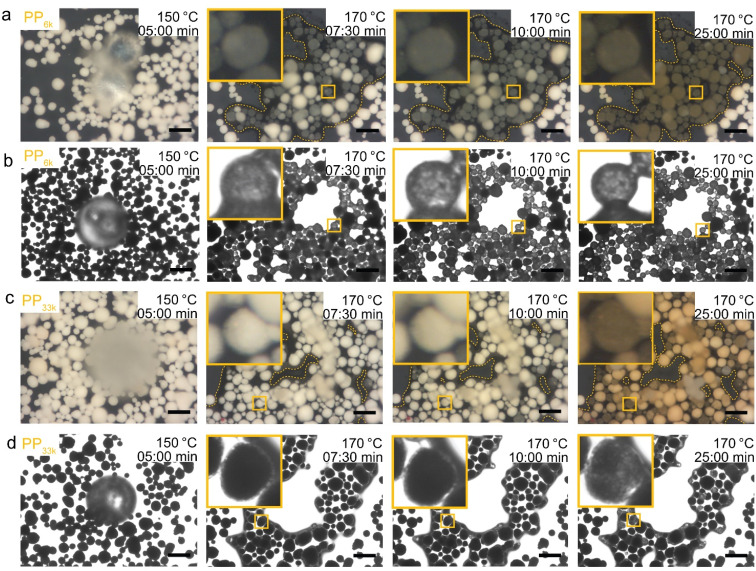
*In situ* optical microscopy of low *M*_w_ PP interaction with FCC-catalyst. The sample was heated under N_2_ flow at 10 °C min^−1^ to 170 °C and the temperature consecutively held. Timer started upon reaching of 100 °C. (a and c) PP_6k_ and PP_33k_ with illumination from the top. Outlines drawn to illustrate spread of polymer melt. (b and d) PP_6k_ and PP_33k_ with illumination from the below. Images were acquired in greyscale, exposure time was fixed to 200 ms. Scale bars: 100 μm.

Optical microscopy can also be utilized to qualitatively compare pore transport of polymers by leveraging refractive index matching. When PP_6k_ was heated over FCC particles to 170 °C at 10 °C min^−1^ and the temperature held, a large share of the catalyst particles turned dark upon contact with the PP melt (Fig. 7a, see ESI† for full videos). The colour change is comparable to the effect observed when paper is brought into contact with oil: the air between the individual paper fibres is replaced by oil, whose refractive index more closely matches the one of the paper fibres than the one of air. Therefore, the paper appears darker, as less light falling on it is scattered, but also turns the paper more translucent for the same reason. An increase in translucency is also observed for FCC-PP system, when the sample is imaged in transmission mode (Fig. 7b). As the sample is held at 170 °C, more and more particles turn dark or more translucent, suggesting uptake of polymer. Increased translucently can also be observed at room temperature, when the catalyst is brought into contact with immersion oil, which can enter the particle interior (Fig. S15†). At sufficiently long reaction times, cracking products begin to turn the catalyst particles slightly yellow. If the experiment is conducted with a higher molecular weight polymer like PP_33k_, the process is significantly slower (Fig. 7c and d, see ESI† for full videos). Once 170 °C is reached, almost none of the particles appeared dark or translucent. Only at extended reaction times some particles appeared to absorb the higher *M*_w_ polymer. It can be therefore concluded that transport of polymer into the particle interior is significantly slower for PP_33k_ than for PP_6k_. This form of refractive index matching by the polymer melt allows to quickly assess whether polymer is entering the catalyst pore system.

### Effect of molecular weight on coking and selectivity

**Fig. 8:**
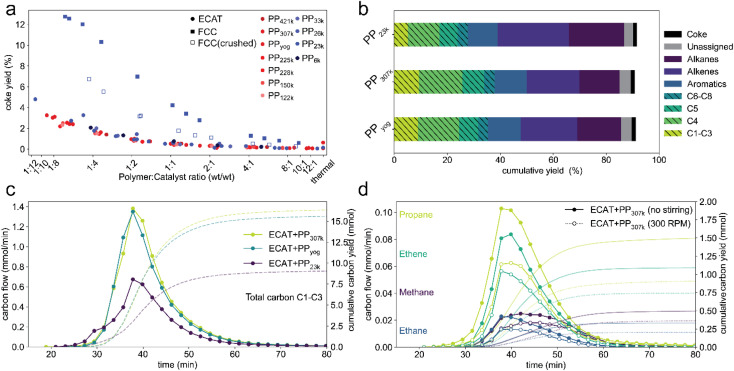
(a) Coke yield determined by TGA for catalytic cracking of PP of various molecular weight using ECAT, FCC-catalyst and crushed FCC-catalyst. (b) Cumulative yields for semi-batch catalytic cracking of 2.5 g of 3 different PP samples using 1.25 g ECAT. Gaseous products (striped fill) were analyzed using online GC, liquid products were identified using offline gas chromatography-mass spectrometry (GC-MS) and quantified using gas chromatography-flame ionization detection (GC-FID) analysis (Fig. S17†). Coke yields were determined by thermogravimetric analysis (TGA) of the spent catalyst materials. Experiments for PP_23k_ were conducted in triplicate, overall gas and liquid yield showed a standard deviation of 2% and 1% respectively. (d) Molar flow and cumulative yield of C_1_–C_3_ products normalized by carbon number measured over time of reaction for cracking of 3 different PP samples. (c) Molar flow and cumulative yield of individual C_1_–C_3_ products excluding propylene normalized by carbon number measured over time of reaction for cracking of PP_307k_ using ECAT without stirring (closed symbols) and stirring at 300 rpm (open symbols).

Mass transport effects might also affect catalyst activity by influencing deactivation *via* coking. A higher coke content has been shown to decrease activity of an FCC catalyst in the catalytic cracking of PP.^44^ In all described TGA experiments, the sample was heated under an oxygen atmosphere after cracking to burn off coke that formed during the reaction. The coke yield, that is, the weight fraction of polymer converted to coke for TGA experiments using PP of various molecular weight as well as FCC-catalyst and ECAT is depicted in Fig. 8. The coke yield decreases with increasing polymer excess in all cases. Coking behaviour is very similar for all molecular weights with a given catalyst, even when comparing PP_6k_ to PP_421k_ for which cracking occurs at vastly different temperatures. This suggests that the described mass transport effects do not affect catalyst deactivation by coking. Coking is significantly enhanced for the fresh FCC catalyst. This can be explained by the high zeolite content of the pristine FCC, while for the ECAT the zeolite is largely degraded. Interestingly, crushing the FCC catalyst significantly reduces coke formation. For the pristine catalyst, gas molecules likely get trapped in the particle interior, increasing residence time and therefore the chance of coke formation. If the catalyst is crushed, diffusion pathways shorten, and the gas does not need to escape through the poorly permeable particle shell. From these results it can be inferred that coke formation does not occur in a single step from plastic but is preceded by formation of smaller molecules. Having established significant differences for catalyst activity when converting different PP samples, the effects of *M*_w_ on selectivity were investigated. For this a series of semi-batch pyrolysis experiments analogous to a prior study^14^ were conducted. PP (2.50 g) and ECAT (1.25 g) were loaded into an autoclave reactor and the temperature was ramped from room temperature to ∼450 °C at 10 °C min^−1^ under constant N_2_ flow. Liquid products were collected in two cold traps held at 0 °C and analysed using gas chromatography (GC) with both mass spectrometry (MS) and flame ionization detector (FID) analysis. Gaseous products up to C_8_ were analysed using on-line GC. The experiment for PP_23k_ was conducted in triplicate (Fig. S16†) and was shown be very reproducible, the standard deviations for overall gas and liquid yield were 2% and 1% respectively. Fig. 7a depicts the overall cumulative yield for the catalytic cracking of PP_23k_, PP_307k_, and PP_yog_. Aromatic yield (∼12%), coke yield (∼1.5%) and mass balance (∼91%) are very similar for all three samples. The similar coke yield for all polymers is consistent with TGA experiments. The deviation of the mass balance from 100% is due to small amounts of liquid products remaining in a cold section of the autoclave and not collected in the condensers. The largest difference observed is in the selectivity towards C_1_–C_4_ gases. For both PP_yog_ and PP_307k_ the yield of C_1_–C_3_ as well as C_4_ is noticeably higher than for PP_23k_. The difference is even more evident when the time resolved formation of C_1_–C_3_ compounds is compared (Fig. 8c). The increased gas formation could be due to over-cracking caused by film diffusion limitations. The diffusion coefficient for short hydrocarbons is in the order of 6 × 10^−9^ cm^2^ s^−1^ in a polyethylene melt,^63^ while its around 0.1 cm^2^ s^−1^ in air.^64^ Therefore it is safe to assume that cracking products will move significantly faster in a gas-filled particle interior rather than travel through the melt. For the higher *M*_w_ polymers, the polymer potentially forms a thick, viscous layer around the catalyst particles. In a cracking event that forms an *e.g.*, C_10_ product, the molecule could therefore not leave the particle easily, as it has to penetrate the viscous polymer film. Instead, the molecule moves back into the pores, leading to over-cracking. For the low molecular weight polymer, the film is less viscous and can be expected to be thinner as it can spread over a larger catalyst surface more easily. The cracking products can leave the active site faster, leading to less over-cracking and a decreased selectivity towards light gases. Evidence for this hypothesis was obtained by comparing a stirred with an unstirred reaction (Fig. 8d). The stirring decreased formation of C_1_–C_3_ gases excluding propylene. This could be explained by a disruption of the polymer film.

The propylene formation is mostly unaffected by stirring (Fig. S18†), as it is largely formed by chain end scission^14^ and therefore can be assumed to be unaffected by the described over-cracking. The effect of stirring on the selectivity indicates that film-diffusion is a limiting factor, though more direct evidence will be required to provide further evidence for this hypothesis. However, C_1_–C_4_ gas formation is undesired, as they are commonly burned in commercial pyrolysis units to heat the overall process and only a limited amount is necessary to supply sufficient heat for the reaction. Furthermore, the individual C_1_–C_4_ components are difficult to separate from each other, requiring energy intensive cryogenic fractionation at commercial scale.^65^

## Conclusions

Based on our observations we propose that catalytic cracking of polypropylene using a fluid catalytic cracking catalysts is inhibited by 3 types of transport limitations.

(i) The high melt viscosity of realistic, high molecular weight polymers severely restricts contact with the external catalyst surface and prohibits effective cracking below 400 °C, while for low molecular weight model polymers cracking can be conducted well below 300 °C. To enable efficient cracking at milder conditions, the viscosity of the melt must be reduced, either by leveraging melt shear-thinning properties or molecular weight reducing pre-treatments, analogous to vis-breaking in oil refining.

(ii) High molecular weight polymers do not enter the pore network of the catalyst below the cracking temperature, while low molecular weight polymers only do so for some particles. While this type of catalyst is designed to process a petrochemical feed that is similar to molten plastic (vacuum gas oil), the pore entries are still too small for efficient transport of the large macromolecules to the particle interior. From the results described here, the exact physical properties determining how well polymer can enter in the pores cannot be identified. Melt viscosity, chain entanglement, steric effects, capillary forces, and low polymer self-diffusion are likely to contribute to different levels depending on the pore diameter considered. However, we expect that these limitations can be overcome by utilizing catalysts with even larger pores and smaller particle sizes. In addition, stronger pre-cracking functionality at the outer particle surface could be implemented to form smaller molecules that can further react in the particle interior.

(iii) Over-cracking due to film diffusion limitations shifts the selectivity toward undesired light gases. To combat this problem, the polymer needs to be well mixed with the particles, and the film needs to be disrupted *e.g.*, by strong stirring.

We believe that the observations described herein can be generalized to a broader range of chemical recycling strategies involving solids catalysts. For example, the long reaction times often required in hydrocracking or hydrogenolysis of polyolefins might be caused by limited catalyst–polymer contact or pore transport limitations. Overcoming these limitations will require redesigning common cracking catalysts to improve pore accessibility, as well as re-thinking traditional approaches to hydrocarbon processing to decrease the melt viscosity. En route to processes that allow catalytic polyolefin conversion at milder conditions and higher selectivities, close attention needs to be paid to the molecular weight of the polymer studied, as using low molecular weight model polymers might lead to overestimation of catalyst activity. Realistically, high molecular weight polymers need to be investigated as well. While this work showcases different types of transport phenomena and how they can be studied, more effort will be required to fully understand which physical properties of both catalyst and polymer are determining mass transport in direct processing of plastic waste using porous heterogeneous catalyst.

## Data availability

All data and python scripts utilized in the manuscript have been uploaded to the YODA repository and are available under https://doi.org/10.24416/UU01-72ZJU1.

## Author contributions

S. R. and I. V. conceptualized the specific research in close consultation with B. M. W., who conceived the overall project and acquired necessary funding. I. V. developed and conducted kinetic modelling. M. J. W. conducted electron microscopy experiments and image segmentation. S. R. conducted the remaining experiments. F. M., E. V. and B. M. W. participated in the discussion of the experiments and related results. The manuscript was written through contributions of all authors. All authors have given approval to the final version of the manuscript.

## Conflicts of interest

There are no conflicts to declare.

## Supplementary Material

SC-014-D3SC03229A-s001

SC-014-D3SC03229A-s002
