# Supplementary material for: Transport limitations in polyolefin cracking at the single catalyst particle level
Source: Chem Sci. 2023 Aug 16;14(37):10068–80. doi: 10.1039/d3sc03229a (PMC10529962; doi:10.1039/d3sc03229a)
Supplement: SC-014-D3SC03229A-s001 [file SC-014-D3SC03229A-s001.pdf]

Electronic Supplementary Material (ESI) for Chemical Science.
This journal is © The Royal Society of Chemistry 2023

Supplementary Information: Mass Transport Limitations in Polyolefin Cracking at the Single Catalyst Particle Level

Materials

Polymers

Tab. S1. Polymer utilized with key properties obtained from both supplier and measurements.

Polymer label	Supplier	Product nr.	Supplier label	Shape	Melting point[a] [°C]	MFR[b] (supplier) [g 10 min^{-1}]	M_n[c] [g mol^{-1}]	M_w[c] [g mol^{-1}]	PDI[d]	Iso-tacticity (IR)[e]
PP_{23k}	Sigma Aldrich	428116	MKCH4322	pellets	158	NA	7300	22700	3.1	91%
PP_{307k}	Sigma Aldrich	427888	MKCD0180	pellets	172	12	47000	306800	6.5	92%
PP_{33k}	Sanyo Chemical		Viscol 330-P	powder	152	NA	11900	32500	2.7	89%
PP_{26k}	Sanyo Chemical		Viscol 440-P	powder	151	NA	9900	26400	2.7	90%
PP_{6k}	Sanyo Chemical		Viscol 660-P	powder	144	NA	2700	6200	2.3	92%
PP_{225k}	LyondellBasell		Moplen HP483R	pellets	167	27	49800	225100	4.5	83%
PP_{420k}	Sigma Aldrich	427861	MKCN5172	pellets	165	4	62300	420800	6.8	91%
PP_{208k}	Sabic		201118tw2	powder	165	31.37	32200	207600	6.4	80%
PP_{150k}	Sabic		201119tw1	powder	164	118.24	22800	149600	6.6	83%
PP_{122k}	Sabic		201119tw2	powder	165	317.75	16400	121600	7.4	83%
PP_{yog}	waste			snippets	166	NA	NA	NA	NA	94%

[a]Determined by DSC (see Fig. S10), [b]Melt flow rate (230 °C/2.16 Kg), only when provided. [c]Determined by GPC (see Fig. S3), [d]Polydispersity Index. NA: Not availible. [e]Determined by infrared spectroscopy from ratio of absorbance at 998 cm^{-1} and 973 cm^{-1} according to Ref.[1]

For thermogravimetric analysis (TGA) experiments, PP_{23k} was lightly crushed using a pistil and mortar to obtained sufficiently small particles. PP_{213k}, PP_{256k} and PP_{340k} were cut using a razorblade. PP_{yog} was taken from a white yoghurt cup. All polymers were provided by the respective manufacturer, with the exemption of Sigma Alrich.

Catalysts

A fresh FCC catalyst (FCC-cat) as well as an equilibrium catalyst (ECAT) were obtained from Albemarle. All catalyst materials were calcined before use by ramping the temperature to 120 °C with 5 °C min^{-1}, holding the temperature for 20 min and subsequently ramping the temperature to 550 °C using a ramp of 10 °C min^{-1} and holding the temperature at 550 °C for 5 h. For experiments with crushed catalysts, the catalyst particles were crushed for 5 min using a pistil and mortar. Summary of surface Aarea and pore characterization of the catalysts is given in Tab. S2, full isotherms are depicted in Fig. S6. Acid site characterization of ECAT and FCC can be found in prior work utilizing the same catalyst materials.[2]

Tab. S2. Characterization of catalysts by N_2 physisorption. Isotherms are depicted in Fig. S6.

Catalyst	BET surface area [m^2 g^{-1}][a]	t-plot micropore volume [cm^3 g^{-1}]	Total pore volume (p/p^0 = 0.99)	Mesopore volume [cm^3 g^{-1}][b]
ECAT	201	0.04	0.28	0.23
ECAT (crushed)	180	0.04	0.25	0.21
FCC	266	0.07	0.19	0.12
FCC (crushed)	270	0.08	0.20	0.12

[a]Calculated using BETSI extended Rouqerol criteria.[3] [b] calculated as $V_{meso} = V_{total}\text{-}V_{micro}$

Experimental procedures

Thermogravimetry

Thermogravimetric analysis (TGA) experiments were carried out using a Perkin Elmer 8000 TGA instrument equipped with an autosampler. For most experiments, 5 ± 0.5 mg of polymer and the necessary amount of catalyst to reach the P:C ratio of interest were used. The absolute mass of polymer can have a slight effect on T_{max} however for studying large excess of catalyst or polymer, using 5 mg of plastic proved impractical. Therefore, for P:C < 1:6, 2 ± 0.5 mg of polymer were used, and for P:C > 6.5:1, 10 mg of polymer were used. This was kept constant between experiments to ensure small changes in T_{max} can be interpreted meaningfully. Alumina crucibles (Perkin Elmer, Part no. N5370464) were utilized. All crucibles were tared prior to sample loading. The catalyst was loaded into the crucible first, and evenly distributed on the bottom of the crucible. Next the polymer was added on top of the catalyst, either as powder as received, as powder or small grains obtained by grinding PP_{23k} in a pistill and mortar, or as larger grains obtained by cutting large peletts of the high M_w polymers using a razorblade. The loaded crucible was then lightly tapped on a solid surface to make plastic sink in the catalyst.

The temperature was first ramped from 50 °C to 600 °C at 10 °C min^{-1} under 45 ml min^{-1} N_2 flow, then cooled down to 50 °C. Then the sample was heated to 800 °C at 10 °C min^{-1} under 45 ml min^{-1} O_2 flow. The precise P:C ratio was determined from the TGA data, as the microbalance in the instrument is more precise and the catalyst looses moisture upon heating. The mass of catalyst was determined as the measured weight after completed burnoff. The amount of polymer was determined by subtracting the mass of catalyst from the weight measured after most of the water evaporated at 150 °C.The mass of coke is determined by substracting the mass of the catalyst from the sample mass at the end of cracking.

m(catalyst) = m(800 °C, O_2)

m(polymer) = m(150 °C, N_2) – m(catalyst)

m(coke) = m(600 °C, N_2) – m(catalyst)

The differential thermogravimetric analysis (DTGA) data were calculated by dividing changes in mass by changes in temperature. T_{max} was determined as the temperature at which the

DTGA was highest. Comparing the temperature at which 50% of the polymer was converted to gas (T_{50}, another common metric for similar experiments) gave similar results. For plotting, the DTGA was smoothed by using a rolling average over 15 datapoints (corresponding to less than 1 °C temperature change).

Kinetic modelling

Kinetic modelling was conducted using a custom python script drawing on prior work by Marcilla *et al.*[4] In short, simulated weight loss profiles for different catalyst concentrations were calculated using the kinetic equations described in the main manuscript for a dataset containing multiple P:C ratios. Temperature was adapted at a 10 °C/min ramp as with the experiment. The kinetic parameters were optimized by minimizing the deviation from the TGA data using data from Ref.[4] as initial guesses.

In-situ optical microscopy of catalytic cracking

For in-situ optical microscopy, the cracking reaction was conducted in a Linkam FTIR600 Cell. A glass cover slip was placed on the heating element. A small amount of FCC-cat was added, resulting in a layer of catalyst particles in the region of interest. A small piece of plastic (from crushed pellets or cut using a razorblade) was placed on the catalyst bed. The temperature was ramped at 10 °C min^{-1} to 500 °C under 100 ml min^{-1} N_2 flow and images were acquired every minute or 30 s starting at 100 °C using a Zeiss Axio Zoom, v16 optical microscope equipped with a camera. For experiments shown in Fig. 7 in the main manuscript the temperature was kept constant after reaching 170 °C. For images aquiered using transmission iluination, the exposure time was fixed to 200 ms. Movies were generated from the images using a custom python script.

Electron microscopy of PP-FCC composites

The internal morphology of PP-embedded samples was assessed with a FEI Helios NanoLab G3 UC scanning electron microscope (SEM) following procedures from literature.[5,6] The samples were dispersed onto a double-sided adhesive, conductive carbon tape, which was attached to an aluminum SEM stub. To improve the conductivity of the sample, a Pt coating of ~10 nm thickness was applied with a Cressington 208HR sputter coater prior to focused ion beam (FIB)-SEM. All particles were cut perpendicular to the surface of the stub at a stage tilt angle of 52 °. The cross-sectional images were acquired in backscattered electron (BSE) mode at 2 kV and 0.1 nA using a through the lens detector (TLD) and an immersion lens.

Semi-batch catalytic cracking experiments

Semi-batch catalytic cracking experiments were conducted following an adapted procedure described previously.[2] 2.50 g of PP (pellets for PP_{23k} and PP_{307k}, stripes ca. 1 cm in size for $PP_{yog,}$ cut from a yoghurt cup) and 1.25 g of ECAT were sequentially loaded into a 50 mL Parr autoclave reactor. The autoclave was slightly shaken to distribute the catalyst equally. A 30 ml min^{-1} flow of N_2 was sent through the reactor and served as carrier gas for online gas chromatography (GC) analysis of gasous products formed. The condansable products are collected in 2 icewater-cooled condensers (Fig. S1). The gas lines between reactor and

condenser were kept at 300 °C. The pressure in the system was near ambient (1.1 atm) After the reactor has been purged with N_2 until no O_2 was detected on the on-line GC, the temperature was ramped to ~450 °C at ~10 °C min^{-1} and held for 1 h using a calibrated power profile, as described previously.[2] After the experiment was finished, the condensers were weighted to determine liquid yield. The coke yield was determined by TGA of the spent catalyst. On-line GC analysis of products was conducted analogously to a previously published procedure.[2]

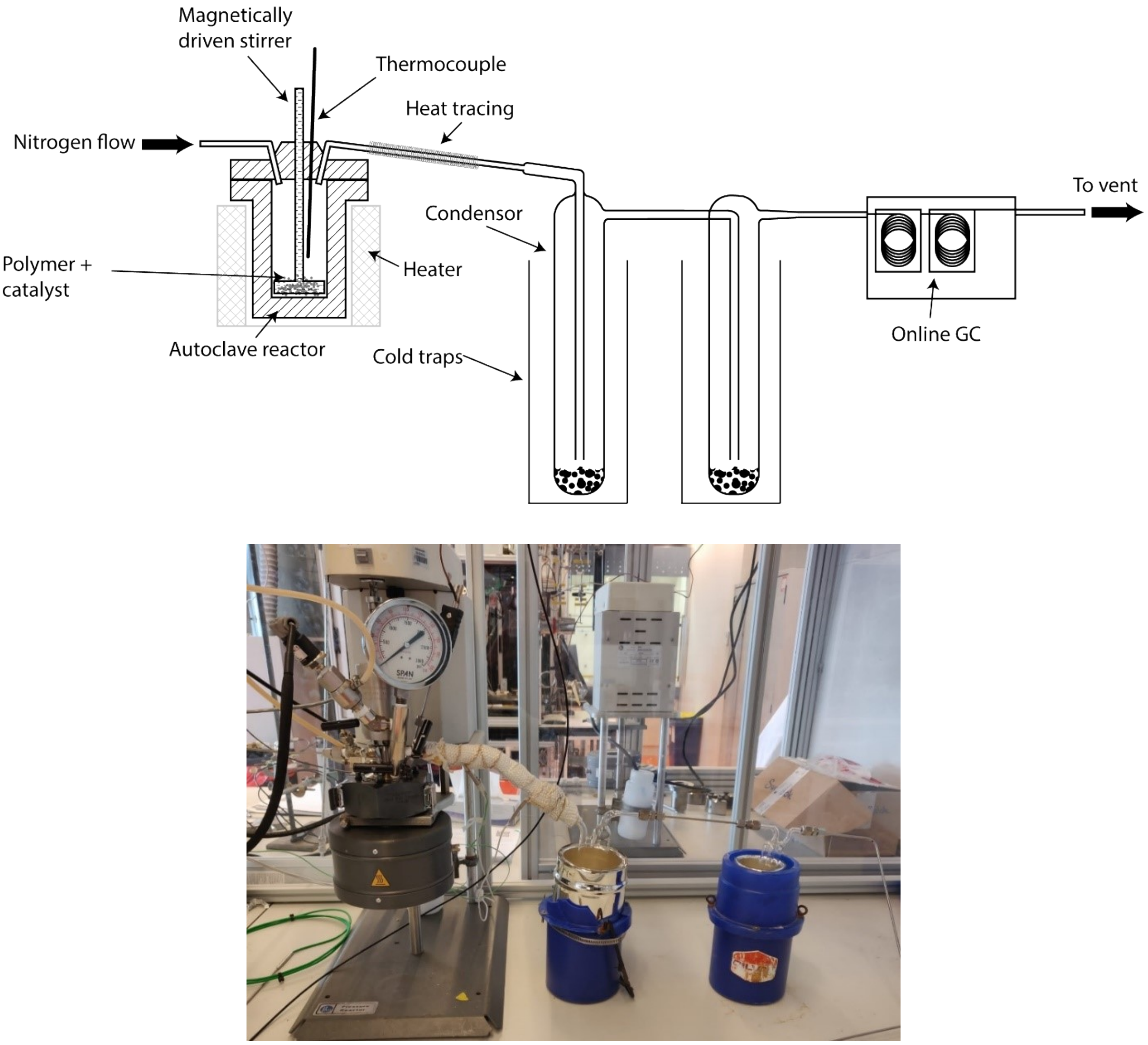

Fig. S1. Top: Schematic representation of reactor system utilized for semi-batch catalytic cracking of PP. Bottom: Photograph of the setup.

Condensable product characterization

To identify compunds in the condesable products, gas chromatography combined with mass spectrometry (GC-MS) was performed on a Shimadzu GC-2010 instrument equipped with an inert 5% phenylmethyl polysiloxane Agilent VF-5ms column and a Shimadzu GCMS-QP2010 MS. The condensed oil was dissolved and diluted to a 1:100 ratio with dichloromethane. The

split ratio was set to 100, with an injection volume of 1 µl at a temperature of 265 °C. The reaction products were assigned using the Shimadzu GCMS Postrun Analysis software searching the NIST/EPA/NIH Mass Spectral Database (NIST 11). If no satisfying match was found, the product was labeled unassigned. This yieled a product table containing retention times of the peaks in the chromatogram and the respective library match if found. To quantify the products, GC combined with FID was utilized. A Varian 430-GC same column as the GC-MS was used. The split ratio was set to 50 with an injection volume of 1 µl at a temperature of 265 °C.

Using the same column for GC-MS and GC-FID allowed the use of identification from GC-MS for the quantification through GC-FID, assuming that the area fraction corresponds to the mass fraction in the liquid product. This assumption is justified as the signal of the FID mostly depends on the carbon content of the compound for hydrocarbons. The mass fraction of carbon atoms is a good approximation for the total mass fraction if only hydrocarbons are investigated.

As the chromatograms of both instruments don't match perfectly, they had to be aligned. For this the MS chromatogram was shifted and stretched so that retention times of peaks aligned with the same peak on the FID chromatogram. This was achieved using a custom python script. In short: Both chromatograms were plotted together and peaks were annotated with their respective retention times. Then, multiple estimated linear functions were applied to the MS-chromatogram to shift it to rougly match the FID-chromatogram, the peak annotations were set to display the unshifted rention time. For a more precise match, the original retention times of the alligned peaks were read out, and used to construct linear functions. Once a good allignment of MS and FID chromatograms was achieved, the same shifting funtions were applied to the rentention times in the product table, which was then used to asign products to peaks in the FID. FID integration including a baseline substraction and normalization was achieved using a customized python script. The same script was used to classify the compound as either aromatic if the name contained 'Benzene', 'Toluene', 'Naphthalene', 'Chamazulene', 'Azulene', 'Xylene' or 'Cumene'; as alkene if the name contained 'ene' and as alkane if the name contained 'ane'.

Differential scanning calorimetry

Differential scanning calorimetry (DSC) was conducted using a Mettler Toledo DSC 3 STARe system. 5 ± 1 mg polymer was heated in aluminum sample pans from -60 °C or -30 °C to 250 °C, at 10 °C min^{-1} then cooled back down at the same rate, and heated again to 250 °C. The melting point was determined as the first peak in the thermogram of the first heating cycle.

Supplementary results and discussion

S1: Calculation of polymer chain length

The weight-averaged polymer chain length was estimated according to the following procedure based on strongly idealized, non-interacting chains.[7] The mean-square end-to-end distance was calculated using

$$\langle R^2 \rangle = C_\infty n l^2$$

Where C_∞ is the characteristic ratio defined by Flory as the ratio of the actual unperturbed mean-square end-to-end distance $\langle R^2 \rangle$ and that of a freely jointed chain nl^2. For isotactic polypropylene $C_\infty = 6.15$. n is the average number of bonds in the polymer, calculated by dividing the average molecular weight M_w of polypropylene by the mass of a repeating unit and multiplying the resulting degree of polymerization by 2 since each propylene unit corresponds to 2 backbone bonds, and thus with $M(C_3H_6) = 42.08\frac{g}{mol}$ and

$$n = \frac{23{,}000\frac{g}{mol}}{42.08\frac{g}{mol}} \cdot 2 = 1093$$

and l is the average bond length in the polymer and is 1.54 Å

The random coil size was calculated as the root mean square end-to-end distance becomes:

$$R_{rms} = \sqrt{\langle R^2 \rangle} = 126.3\ \text{Å} \approx 13\ nm$$

And the length of the fully extended chain:

$$R_{max} = nl\cos\left(\frac{\theta}{2}\right) = n\ \cdot 1.54\ \text{Å}\ \cdot \cos\left(\frac{68°}{2}\right) = 1395.6\ \text{Å} \approx 140\ nm$$

with θ being the torsion angle of the backbone bonds. Extended chain lengths and coil sizes were calculated analogously for the other PP samples.

S2: Polymer characterization by GPC

Molecular weight distributions were determined by high temperature gel permeation chromatography (HT-GPC), using a Polymer-Char GPC-IR instrument. Molecular weight was calibrated using Polystyrene standards. Due to insufficient solubility, the molecular weight for PP_{yog} could not be determined using this method. To estimate it, an approximate molecular weight can be calculated from the melt flow rate (MFR).[8] Using a MFR of 5.3 g min^{-1} for a PP yoghurt cup from published literature,[9] a M_w of ~334,000 can be estimated. To estimate the molecular weight of commercial PP homopolymer used in packaging, the highest and lowest MFRs (120 g/10 min and 0.5 g/10 min at 230 °C/2.16 kg) for the 'Moplen' polymer by Lyondellbasell were used.[10] Application was filtered by 'Containers' and 'Caps & Closures'.

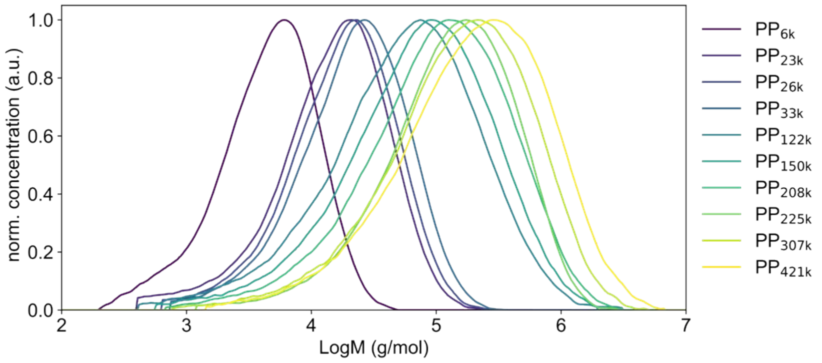

Fig. S1. HT-GPC chromatograms of PP analyzed.

S3: Justification of TGA approach

It is important to note that non-isothermal TGA only allows to study the apparent kinetics of the cracking reaction due to potential mass/heat transport limitations as well as different chemistries occurring at different temperatures, as opposed to the intrinsic kinetics of the reaction.[11] Nevertheless, significant differences in apparent activity obtained from measurements at identical conditions still allow to make conclusions e.g. regarding the accessibility of two different catalysts.

Reproducibility: As can be seen in Fig. 2 of the main manuscript, the polymer to catalyst (P:C) ratio can have a significant effect on the cracking temperature in TGA experiments. To ensure good reproducibility in an experiment aiming at a single P:C ratio, catalyst and polymer would need to be weighted in very precisely, as even slight deviations can cause a significant shift in cracking temperature, especially at low catalyst concentrations. This problem can be overcome by investigating multiple P:C ratios and comparing the trends between sets of measurements. Furthermore, we found that the total amount of polymer also has a slight effect on the cracking temperature. Therefore, the mass of polymer needs to be kept similar in between experiments. This approach allows for high reproducibility, as can be seen in Fig. S2a. In this work, comparisons are never made between two individual datapoints, instead trends over wide ranges of P:C ratios are compared, allowing to draw conclusions with a significantly higher degree of confidence.

Thermal ramp vs. isothermal experiments: While isothermal experiments are a simpler approach to access reaction kinetics more directly, they make comparisons between high and low M_w polymers difficult, as the cracking occurs in vastly different temperature regimes for both samples (see Fig. 6 main manuscript).

Heat transfer limitations: At the used heat rate of 10 °C min^{-1} heat transfer effects play only minor role, as can be demonstrated by conducting the experiment at varying heating rates shown in Fig. S2b. T_{max} for 5 °C min^{-1} is only marginally lower than for 10 °C min^{-1}, while at 20 °C min^{-1} T_{max} is noticeably higher, suggesting heat transfer limitations.

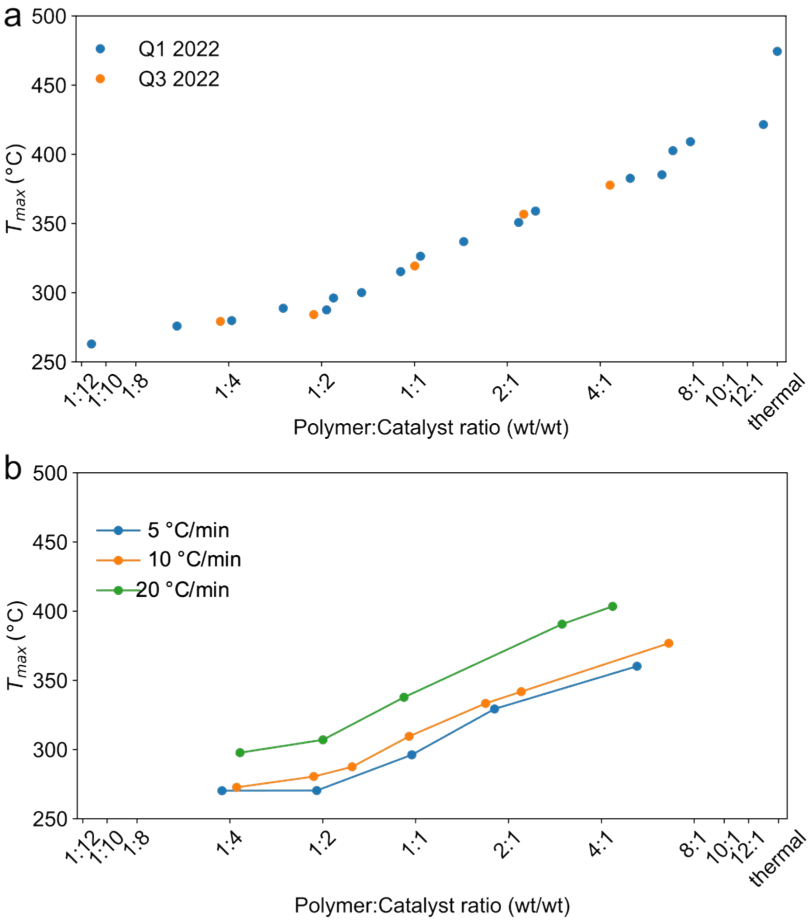

Fig. S2. (a) T_{max} for cracking of PP_{23k} using ECAT recorded 6 months apart on separate calibrations of the TGA instrument. (b) T_{max} for cracking of PP_{23k} using ECAT at 3 different heating rates.

S3: Kinetic modelling

Table S3: Parameters determined by kinetic modelling**.**

Parameter	ECAT + PP_{23k}	FCC + PP_{23k}	FCC_{cr} + PP_{23k}	FCC +PP_{6k}	FCC +PP_{26k}	FCC +PP_{33k}
n_1	0.52	0.09988	0.09988	0.135532	0.136783	0.134411
n_2	1.81	3.517897	3.517897	1.079467	1.105183	1.448927
n_3	0.78	0.7352	0.7352	0.96798	0.942682	0.656122
$k_{0,1}$ (min^{-1})	$3.85 \cdot 10^{12}$	$2.29 \cdot 10^{12}$	$2.29 \cdot 10^{12}$	$1.41 \cdot 10^{13}$	$1.44 \cdot 10^{13}$	$1.94 \cdot 10^{13}$
$k_{0,2}$ (min^{-1})	7116.53	5064.23	5064.23	4425.04	4419.656	3973.912
Ea_1 (kJ mol^{-1})	127.48	103.0735	103.0735	109.4175	109.4421	99.75519
Ea_2 (kJ mol^{-1})	52.83	51.46638	51.46638	49.23823	50.81786	51.86664
c	1.87	4.499953	1.548356	4.573481	4.574673	3.943058

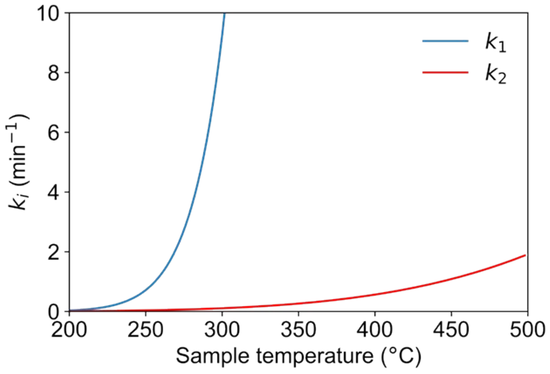

Fig. S3: Evolution of rate constants k_1 and k_2 determined by kinetic modelling with temperature for cracking of PP_{23k} using ECAT.

S4: TGA profiles

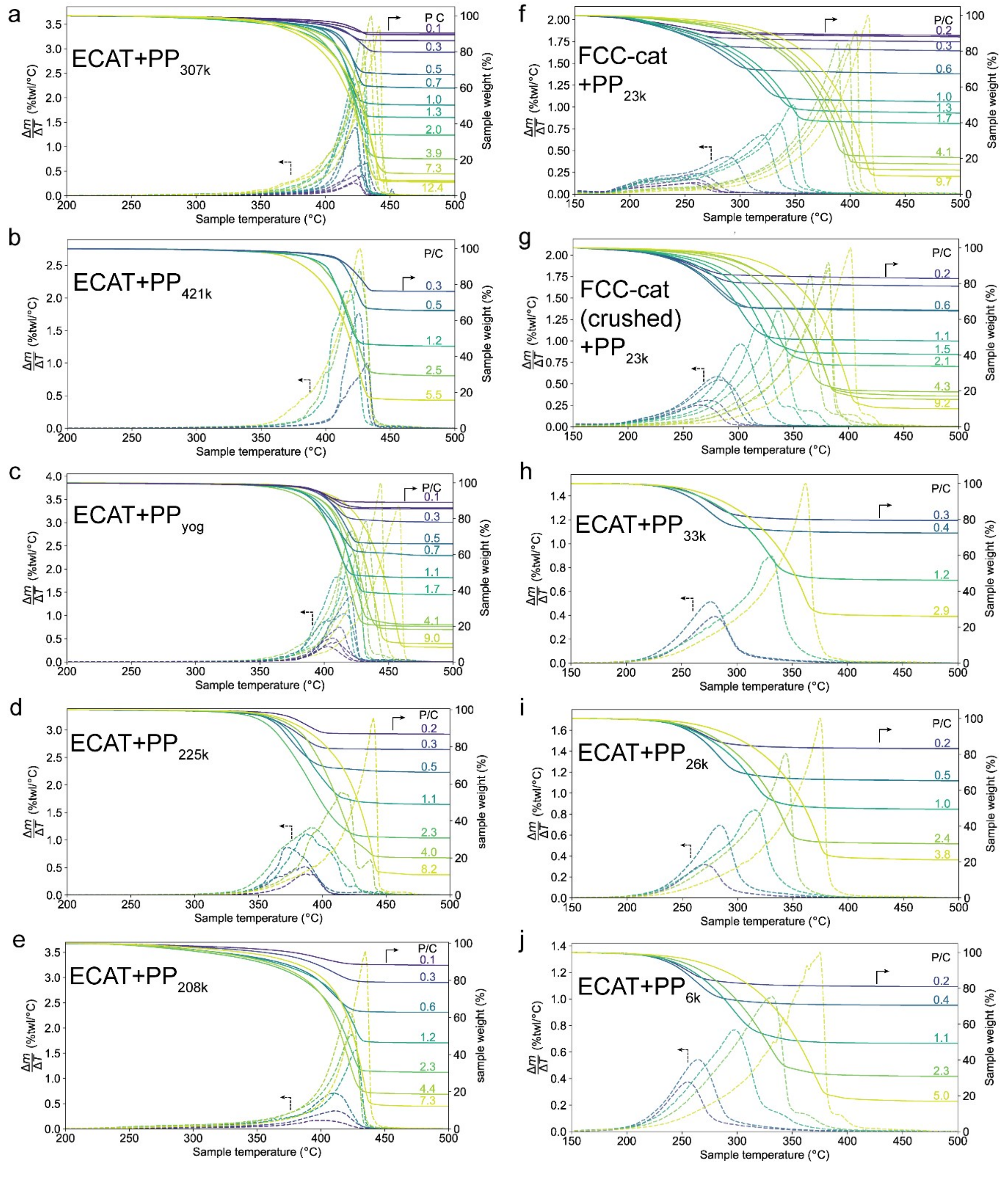

Fig. S4: Thermogravimetric analysis (TGA) profiles for TGA experiments shown in the main manuscript (1 of 2)**.** P/C denotes m(Polymer)/m(Catalyst).

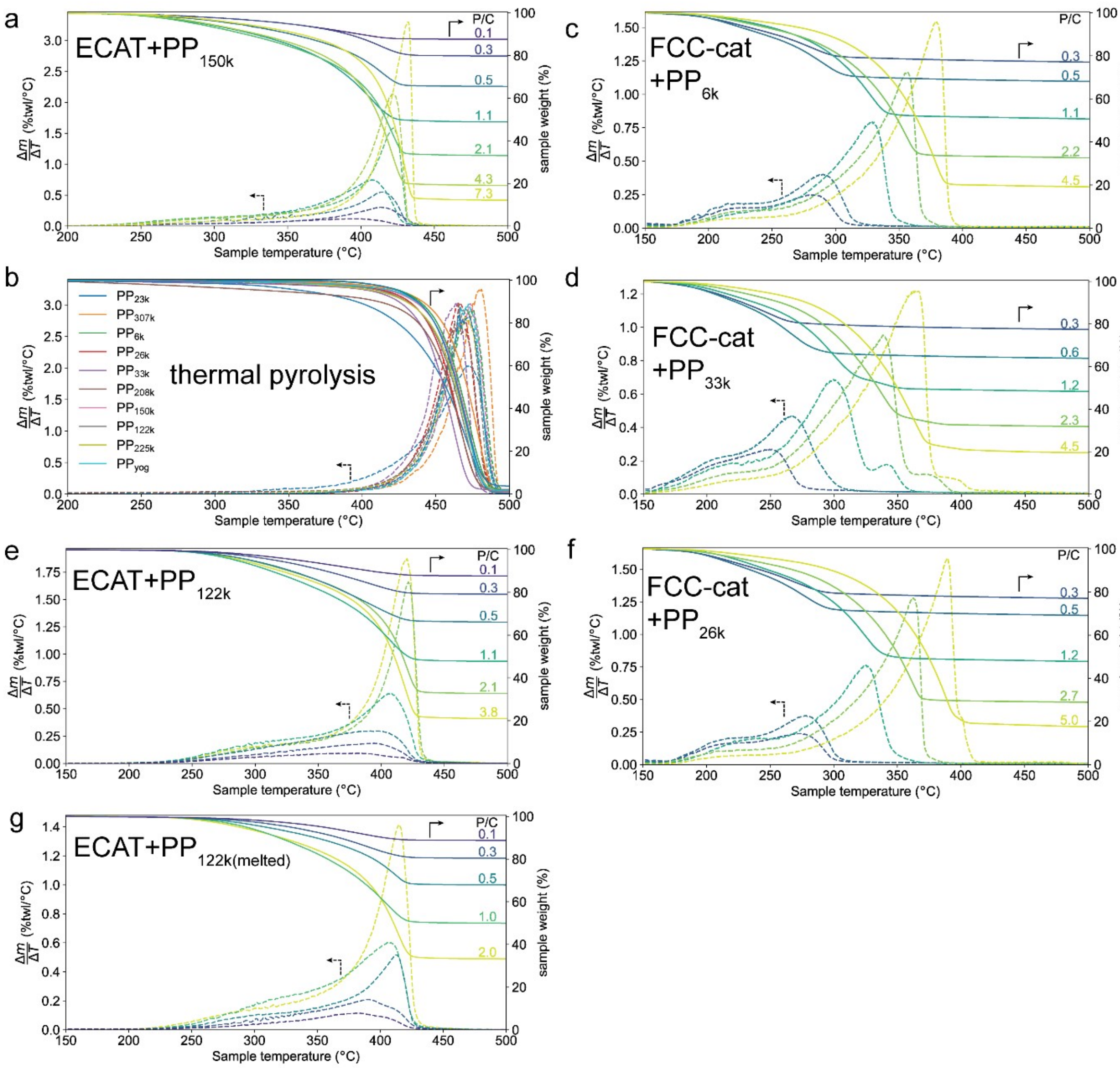

Fig. S5: Thermogravimetric analysis (TGA) profiles for all TGA experiments shown in the main manuscript (2 of 2)**.** P/C denotes m(Polymer)/m(Catalyst).

S5: Catalyst Characterization by N_2 physisorption

N_2 isotherms at 77K were acquired using a 3P Sync 400 physisorption analyzer. BET surface area was determined form the absorption isotherm using BETSI.[3] Micropore volume was determined using the t-plot method. Note that without additional corrections the micropore volume is underestimated for systems that contain both micro and mesopores.[12]

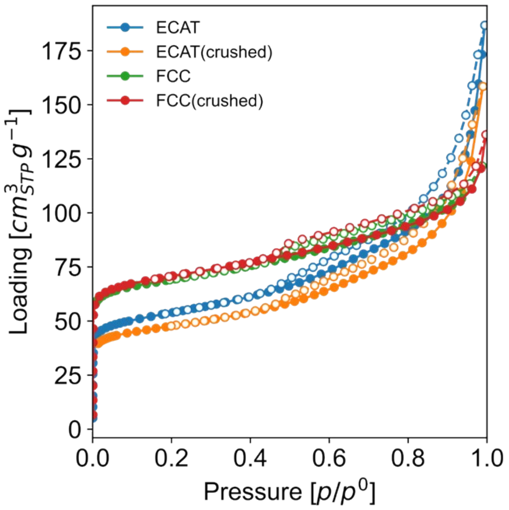

Fig. S6. N_2 isotherms at 77K of catalysts studied. Plotted using pyGAPS.[13]

S6: Effect of sieving and crushing on ECAT/FCC activity

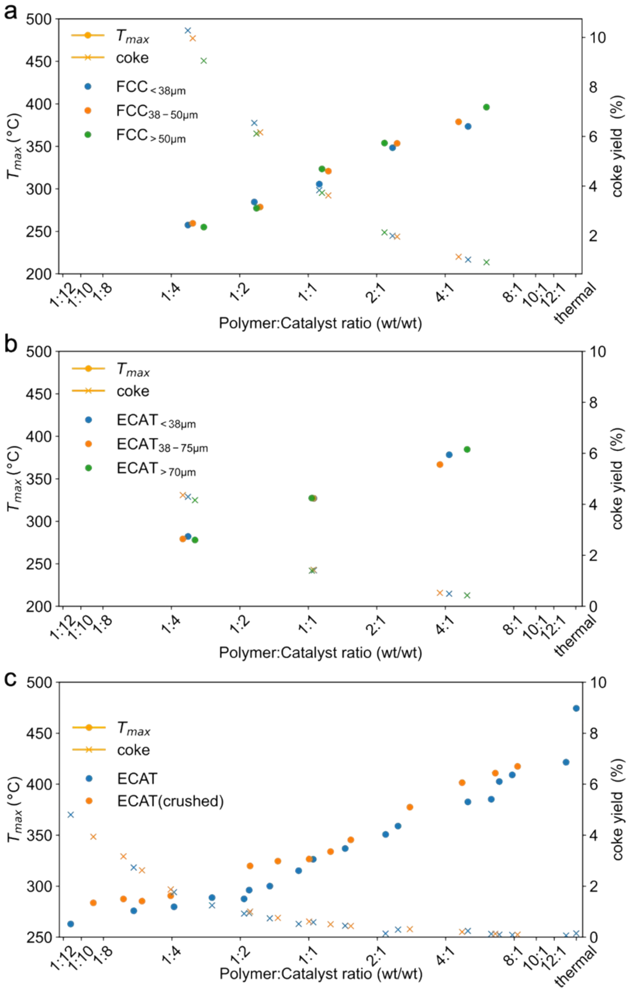

Fig. S7. T_{max} and coke yield for cracking of PP_{23k} using sieving fractions of FCC (a), sieving fractions of ECAT (b) and comparison of crushed ECAT with pristine (c).

S7: FIB-SEM of ECAT

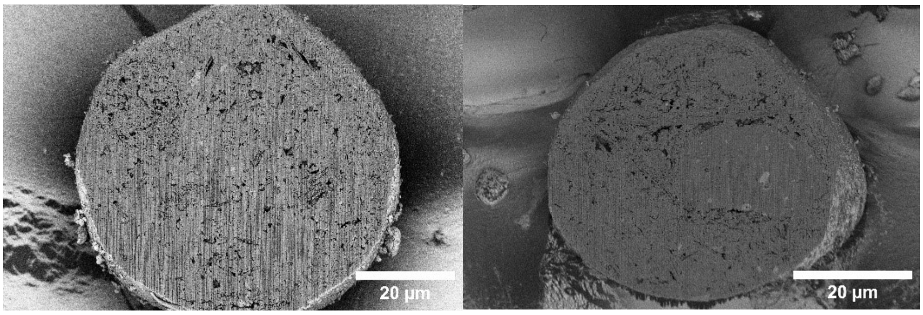

Fig. S8. Scanning electron micrographs of two ECAT particles cut open using a focused-ion-beam.

S8: Determination of melting points by differential scanning calorimetry

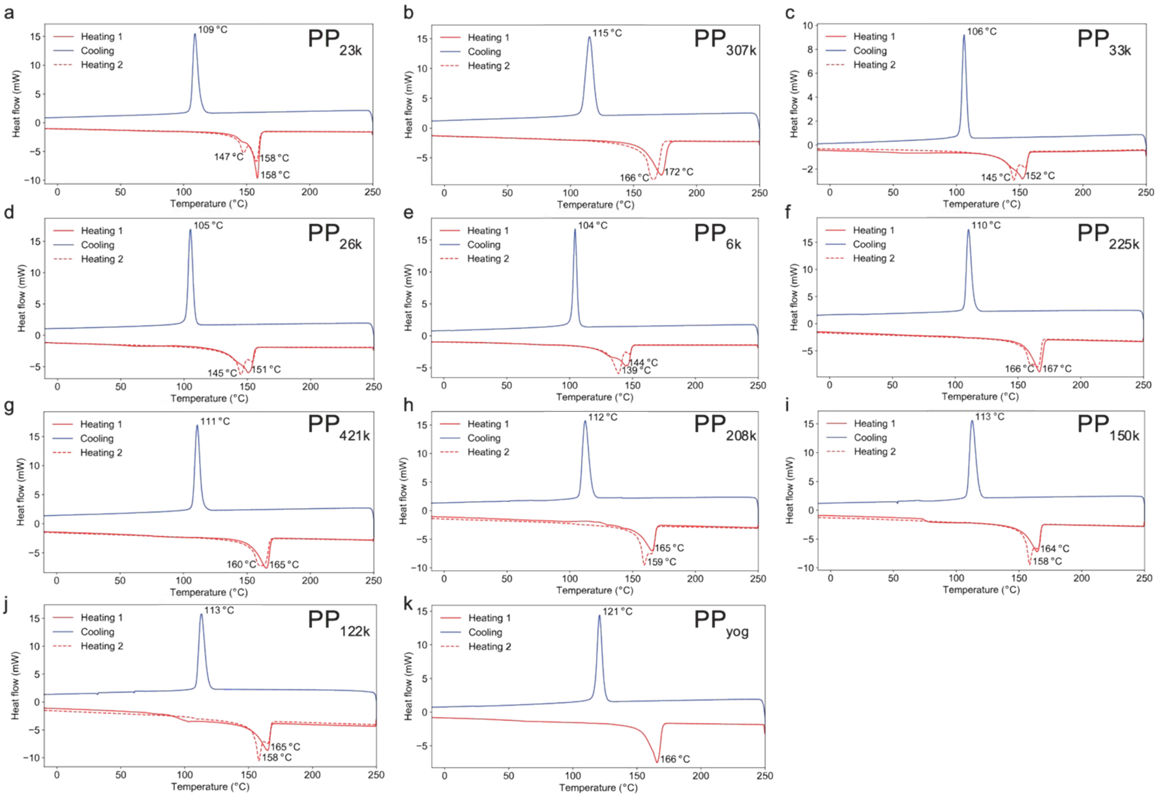

Fig. S9: Differential scanning calorimetry (DSC) thermograms for PP utilized.

S9: Characterization of stereoregularity

Degree of isotacticity was estimated by attenuated total reflectance (ATR)-IR spectroscopy in analogy to Ref.[1] using the ratio of absorbance at 998 cm^{-1} and 973 cm^{-1}. IR spectra of the polymers were acquired using a Perkin Elmer Frontier spectrometer with a diamond ATR module from 4000 cm^{-1} to 450 cm^{-1} at a resolution of 4 cm^{-1} averaging over 32 scans. A ATR correction was applied to the data using the equipment's software. A linear baseline was subtracted in the window of 1020 cm^{-1}-952 cm^{-1}. Isotacticity was determined according to the formula $mm = ((A_{998}/A_{973})+0.15)/1.08$ where A_{998} and A_{973} are the absorbance at the respective wavenumbers. The polymers were not annealed prior to measurement, and therefore the real isotacticity is likely higher. This approach is less accurate than a full stereoregularity analysis using NMR, but shows that all polymers under study are largely isotactic. Normalized IR spectra of the polymers in the relevant region are depicted in Fig. S4

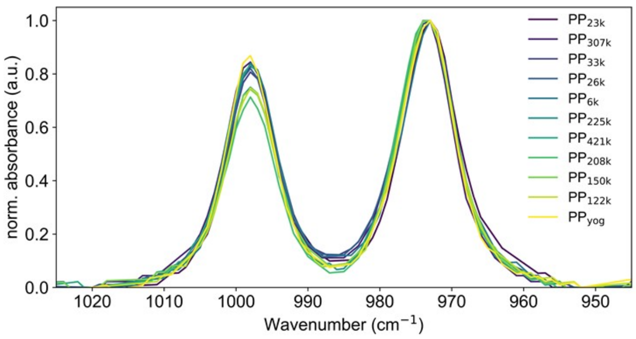

Fig. S10. Normalized, baseline corrected ATR-IR spectra of polymers utilized in this study in the windows utilized for stereoregularity analysis.

S10: Effect of macroscopic polymer grain size

To test for the effect of polymer particle size, PP_{122k} powder was melted down under N_2 atmosphere in a tubular oven (170 °C, 10 min, 10°C min^{-1}). From the resulting mass, polymer grains at least 10x larger than the powder grains were cut out using a razorblade. TGA experiments showed the cracking temperature is not affected by macroscopic grain size.

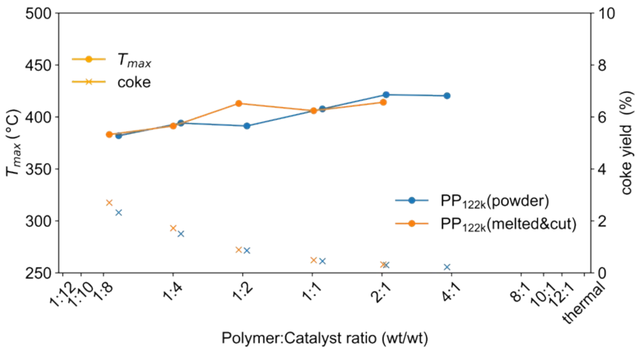

Fig. S11. T_{max} and coke yield for cracking of PP_{122k} powder and grains obtained by melting under N_2 atmosphere using ECAT. Full TGA profiles are given in Fig. S5.

S11: *In-situ* optical microscopy of cracking of additional polymers

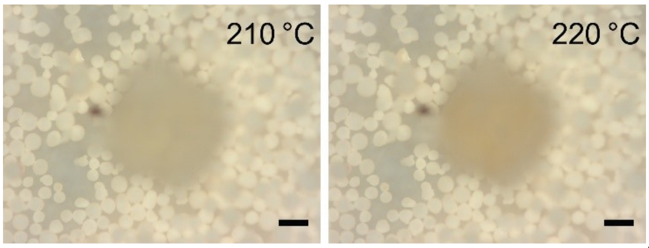

Fig. S12. *In-situ* optical microscopy of P_{307k} cracking over FCC showing color change at 220 °C. Scale bars: 100 µm.

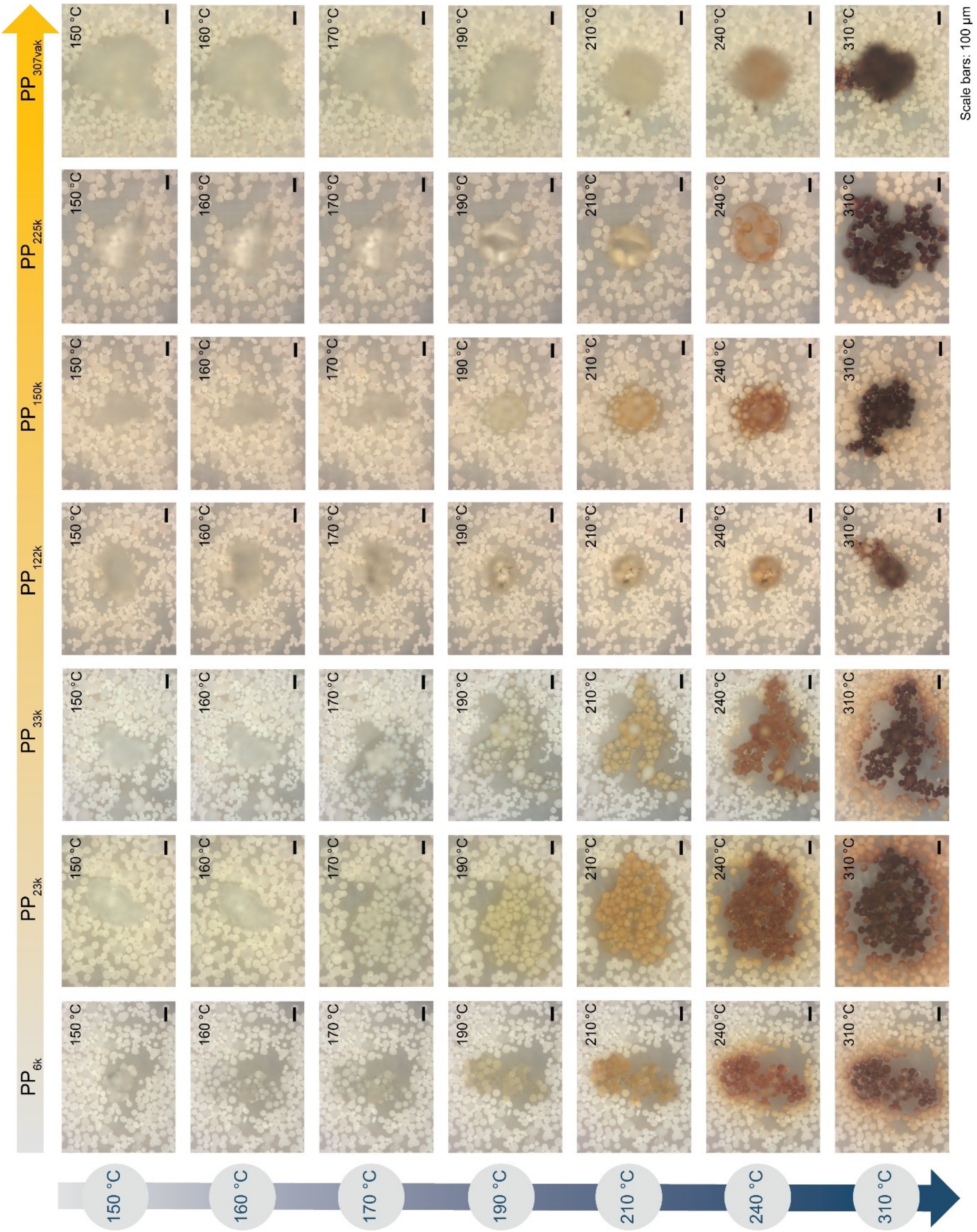

Fig. S13: In-situ optical microscopy of polypropylene (PP) cracking using FCC catalyst. Individual polymer grains were placed on a bed of FCC catalyst and heated at 10 °C min^{-1} under N_2 flow in a Linkam stage. Images were acquired every minute.

S12: Scaling of viscosity with molecular weight and temperature

Viscosity of polypropylene melts scales with molecular weight according to equation 7 in the main manuscript. The relative zero-shear viscosity $\eta_r(MWR)$ of PP melts of different molecular weight ratios MWR can then be then be estimated:

$$\eta_r(MWR) = \frac{\eta(M_{w,1})}{\eta(M_{w,0})} = \left(\frac{M_{w,1}}{M_{w,0}}\right)^{3.4}$$

The temperature-viscosity relationship for PP melts can be described using a shift factor a_T following an Arrhenius-type equation,[14] with $\eta(T)$ as the viscosity at a given temperature, η_0 describing the viscosity at a reference temperature, the value of B is dependent on the chosen reference temperature, E_a being a shift factor 'activation energy', R the gas constant, and T the absolute temperature.

$$\frac{\eta(T)}{\eta_0} = a_T(T) = B \cdot e^{\left(E_a/RT\right)}$$

Mendelson determined the $a_T(T)$ for PP of viscosity-averaged molecular weight of

$M_v = 2.2 \cdot 10^5$ g mol^{-1} over a temperature range of 175 °C – 260 °C using 200 °C as reference temperature:[14]

$$log(a_T(T)) = -4.65 + (2.20 \cdot 10^3) / T$$

Using both relationships, the effect of both M_w and temperature on the melt viscosity can be visualized. It has to be noted that beyond 260 °C polymer degradation leads to a deviation from the temperature scaling shown here (see main manuscript).

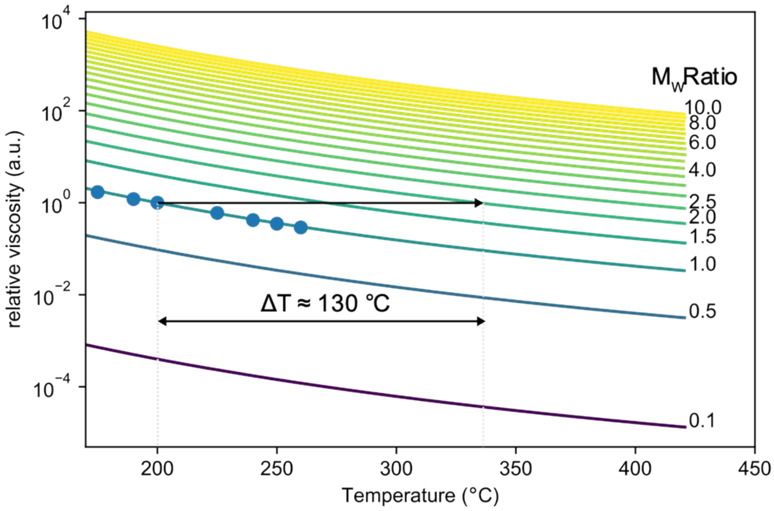

Fig. S14: Scaling of PP melt viscosity for different molecular weight ratios and temperature based on Ref.[14] and Ref.[15] Datapoints shown adapted from Mendelson.[14]

S13: Optical microscopy of FCC brought into contact with immersion oil

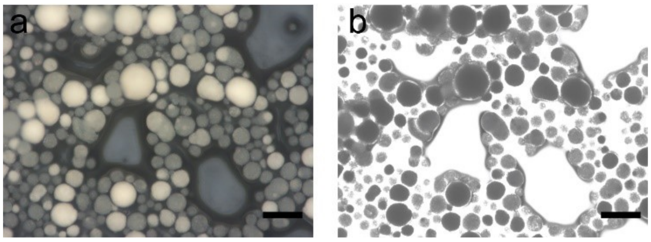

Fig. S15: Optical microscopy images of FCC catalyst brought into contact with microscopy immersion oil. Scale bars: 100 µm. a) Image acquired using illumination form the top. b) Image acquired with illumination from the bottom, greyscale, exposure time of 200 ms.

S14: Reproduction of semi-batch reactor experiments

Semi-batch reactor experiments for PP_{23k} were conducted in triplicate. The profile of the gas evolution and cumulative yields are depicted in Fig. S14. The overall gas yield showed a standard deviation of 2%, while the standard deviation for the C_1-C_3, C_4, C_5, C_6-$_8$ and the liquid yields were 2%, 5%, 2%, 3%, and 1% respectively.

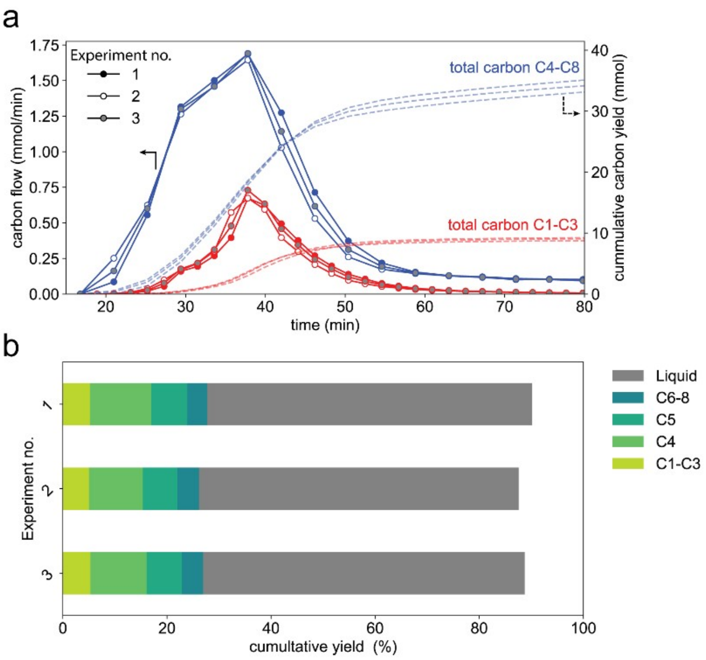

Fig. S16. Reproductions of semi-batch experiments for cracking of PP_{23k} using ECAT. a) Total carbon flow and cumulative yield obtained by on-line GC. b) Overall yield of individual components and liquid.

S14: Chromatograms of liquid products

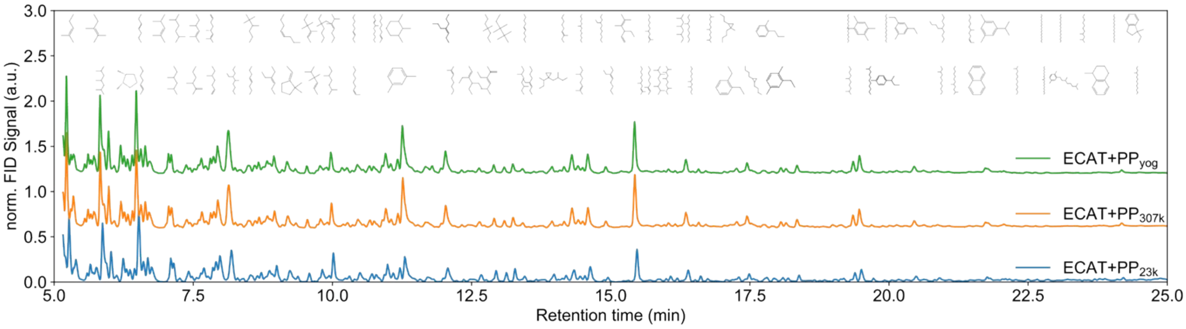

Fig. S17. Gas chromatography-flame ionization detector (GC-FID) chromatograms for catalytic cracking of different polypropylene (PP) samples (PP_{23k}, PP_{256k} and PP_{yog}) using ECAT. Baseline corrected, normalized by chromatogram area and offset for clarity. Species identified by GC-MS are annotated. Some compounds were omitted for clarity.

S15: Propylene formation stirred versus unstirred reaction

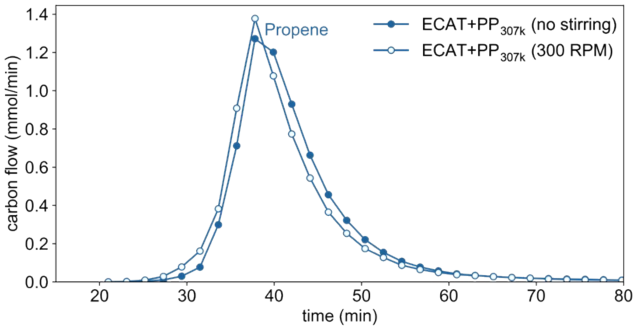

Fig. S18. Propene formation during for semi-batch catalytic cracking of PP_{307k} without stirring (closed symbols) and stirring at 300 round per minute (RPM) (open symbols) as determined by on-line GC.

Supplementary References

1 D. R. Burfield and P. S. T. Loi, *J. Appl. Polym. Sci.*, 1988, **36**, 279–293.

2 I. Vollmer, M. J. F. Jenks, R. Mayorga González, F. Meirer and B. M. Weckhuysen, *Angew. Chem. Int. Ed.*, 2021, **60**, 16101–16108.

3 J. W. M. Osterrieth, J. Rampersad, D. Madden, N. Rampal, L. Skoric, B. Connolly, M. D. Allendorf, V. Stavila, J. L. Snider, R. Ameloot, J. Marreiros, C. Ania, D. Azevedo, E. Vilarrasa-Garcia, B. F. Santos, X. Bu, Z. Chang, H. Bunzen, N. R. Champness, S. L. Griffin, B. Chen, R. Lin, B. Coasne, S. Cohen, J. C. Moreton, Y. J. Colón, L. Chen, R. Clowes, F. Coudert, Y. Cui, B. Hou, D. M. D'Alessandro, P. W. Doheny, M. Dincă, C. Sun, C. Doonan, M. T. Huxley, J. D. Evans, P. Falcaro, R. Ricco, O. Farha, K. B. Idrees, T. Islamoglu, P. Feng, H. Yang, R. S. Forgan, D. Bara, S. Furukawa, E. Sanchez, J. Gascon, S. Telalović, S. K. Ghosh, S. Mukherjee, M. R. Hill, M. M. Sadiq, P. Horcajada, P. Salcedo-Abraira, K.

Kaneko, R. Kukobat, J. Kenvin, S. Keskin, S. Kitagawa, K. Otake, R. P. Lively, S. J. A. DeWitt, P. Llewellyn, B. V. Lotsch, S. T. Emmerling, A. M. Pütz, C. Martí-Gastaldo, N. M. Padial, J. García-Martínez, N. Linares, D. Maspoch, J. A. Suárez del Pino, P. Moghadam, R. Oktavian, R. E. Morris, P. S. Wheatley, J. Navarro, C. Petit, D. Danaci, M. J. Rosseinsky, A. P. Katsoulidis, M. Schröder, X. Han, S. Yang, C. Serre, G. Mouchaham, D. S. Sholl, R. Thyagarajan, D. Siderius, R. Q. Snurr, R. B. Goncalves, S. Telfer, S. J. Lee, V. P. Ting, J. L. Rowlandson, T. Uemura, T. Iiyuka, M. A. van der Veen, D. Rega, V. Van Speybroeck, S. M. J. Rogge, A. Lamaire, K. S. Walton, L. W. Bingel, S. Wuttke, J. Andreo, O. Yaghi, B. Zhang, C. T. Yavuz, T. S. Nguyen, F. Zamora, C. Montoro, H. Zhou, A. Kirchon and D. Fairen-Jimenez, *Adv. Mater.*, 2022, **34**, 2201502.

4 A. Marcilla, A. Gómez, J. A. Reyes-Labarta, A. Giner and F. Hernández, *J. Anal. Appl. Pyrolysis*, 2003, **68**–**69**, 467–480.

5 D. A. M. De Winter, F. Meirer and B. M. Weckhuysen, *ACS Catal.*, 2016, **6**, 3158–3167.

6 M. J. Werny, J. Zarupski, I. C. ten Have, A. Piovano, C. Hendriksen, N. H. Friederichs, F. Meirer, E. Groppo and B. M. Weckhuysen, *JACS Au*, 2021, **1**, 1996–2008.

7 L. J. Fetters, D. J. Lohse and R. H. Colby, in *Physical properties of polymers handbook*, Springer, Berlin, 2007, pp. 447–454.

8 T. Bremner, A. Rudin and D. G. Cook, *J. Appl. Polym. Sci.*, 1990, **41**, 1617–1627.

9 S. Stanic, T. Koch, K. Schmid, S. Knaus and V.-M. Archodoulaki, *Polymers*, 2021, **13**, 1137.

10 Lyondellbasell, Moplen, https://www.lyondellbasell.com/en/products-technology/polymers/trade-name/moplen/.

11 I. Mastalski, N. Sidhu, A. Zolghadr, S. Maduskar, B. Patel, S. Uppili, T. Go, Z. Wang, M. Neurock and P. J. Dauenhauer, *Chem. Mater.*, 2023, **35**, 3628–3639.

12 A. Galarneau, F. Villemot, J. Rodriguez, F. Fajula and B. Coasne, *Langmuir*, 2014, **30**, 13266–13274.

13 P. Iacomi and P. L. Llewellyn, *Adsorption*, 2019, **25**, 1533–1542.

14 R. A. Mendelson, *J. Polym. Sci. Part B Polym. Lett.*, 1967, **5**, 295–299.

15 G. H. Fredrickson, *The theory of polymer dynamics*, Clarendon Press, Oxford, 1996.
